# Supplementary material for: A human-specific motif facilitates CARD8 inflammasome activation after HIV-1 infection
Source: eLife. 2023 Jul 7;12:e84108. doi: 10.7554/eLife.84108 (PMC10359095; doi:10.7554/eLife.84108)
Supplement: Supplementary file 1. — (a) List of primers, gBlocks, and sgRNA sequences. (b) List of antibodies/reagents. (c) Primate CARD8 gene IDs. [file elife-84108-supp1.docx]

**Supplementary File 1**

**Supplementary File 1a: List of primers, gBlocks and sgRNA sequences**

| **Type** | **Target** | **Name** | **Sequence** | **Description** |
| --- | --- | --- | --- | --- |
| primer | human CARD8 | pcDNA3.1_BamHI_mCherry_F | TTGGTACCGAGCTCGGATCCATGGTGAGCAAGGGC | forward primer to clone mC-CARD8 into pcDNA3.1 backbone |
| primer | human CARD8 | pcDNA3.1_EcoRI_CARD8_Cterm_R | GCTGGATATCTGCAGAATTCTTACAAATTCTGCTGTCTAAGATAG | reverse primer to clone mC-CARD8 into pcDNA3.1 backbone |
| primer | human CARD8 | CARD8_cpz_F60L | GGAATTTTTCTTCAGGCTGAGGC | forward primer for T>C mutation in CARD8 introducing a phenylalanine to leucine mutation |
| primer | human CARD8 | CARD8_cpz_F60L | AGCCTGAAGAAAAATTCCAGTTTTTGTGTAT | reverse primer for A>G mutation in CARD8 introducing a phenylalanine to leucine mutation |
| primer | human CARD8 | CARD8_OWM_F60S | GGAATTTTTTCTCAGGCTGAGGC | forward primer for T>C mutation in CARD8 introducing a phenylalanine to serine mutation |
| primer | human CARD8 | CARD8_OWM_F60S | AGCCTGAGAAAAAATTCCAGTTTTTGTGTAT | reverse primer for A>G mutation in CARD8 introducing a phenylalanine to serine mutation |
| primer | human CARD8 | CARD8_F60A_internal_F | GGAATTTTTGCGCAGGCTGAGGC | forward primer for TTT>GCG mutation in CARD8 introducing a phenylalanine to alanine mutation |
| primer | human CARD8 | CARD8_F60A_internal_R | AGCCTGCGCAAAAATTCCAGTTTTTGTGTAT | reverse primer for AAA>CGC mutation in CARD8 introducing a phenylalanine to alanine mutation |
| primer | mCherry | mCherry_F | ATGGTGAGCAAGGGCGAG | forward primer to amplify mCherry-CARD8 |
| primer | human CARD8 | CARD8_Cterm_R | TTACAAATTCTGCTGTCTAAGATAGGACAC | reverse primer to amplify CARD8 |
| primer | human CARD8 | CARD8_seq_internal_F | ATGGAAAAAAAGGAGTGTCC | forward primer for sequence verifying CARD8 construct |

**Table S1 (continued): List of primers, gBlocks and sgRNA sequences**

| **Type** | **Target** | **Name** | **Sequence** | **Description** |
| --- | --- | --- | --- | --- |
| primer | human CARD8 | CARD8_geno_F | GATGTTGCAGTGAGCCAAGA | forward primer for verifying CARD8 KO at sgRNA site |
| primer | human CARD8 | CARD8 _geno_R | CGTCTCACTGCTGTTGTGGT | reverse primer for verifying CARD8 KO at sgRNA site |
| primer | human CARD8 | CARD8_LKO_Sfil_Kozak_F | AAAAGGCCGAGAGGGCCGAATTCGCCACCATGGAAAAAAAGGAGTGTCC | forward primer for cloning CARD8 into pLKO vector with Kozak sequence |
| primer | human CARD8 | CARD8_LKO_linker_Myc_Sfil_R | AAAAGGCCAGAGAGGCCCTAGAGATCCTCTTCTGAGATGAGTTTTTGTTCGCTAGCTGCCGCTCCGCTTCCCAAATTCTGCTGTCTAAGATAGGACAC | reverse primer for cloning CARD8 into pLKO vector with Myc tag |
| primer | chimpanzee CARD8 | NotI_linker_cpz_CARD8_F | AAAAGCGGCCGCAATGGAAAAAAAGGAGTTTCC | forward primer for cloning chimpanzee CARD8 into pcDNA3.1 vector |
| primer | chimpanzee CARD8 | CARD8_cterm_NotI_pcDNA3_R | CTCTAGACTCGAGCGGCCGCCACTGTGCTGGATATCTGCAGAATTCTTACAAATTCTGCT | reverse primer for cloning chimpanzee CARD8 into pcDNA3.1 vector |
| primer | chimpanzee CARD8 | CARD8_LKO_Sfil_Kozak_cpz_CARD8_F | AAAAGGCCGAGAGGGCCGAATTCGCCACCATGGAAAAAAAGGAGTTTCC | forward primer for cloning CARD8 into pLKO vector with Kozak sequence |
| primer | CARD8 | pLKO_check_F1 | GGAGGTCTATATAAGCAGAGCTCTCCC | forward primer to amplify insert in pLKO vector |
| primer | CARD8 | pLKO_check_R1 | CTACTATTCTTTCCCCTGCACTGTACCC | reverse primer to amplify insert in pLKO vector |
| primer | CARD8 | pLKO_check_F2 | CAGTGATAGAGATCTCCCTATCAG | forward primer to sequence insert in pLKO vector |
| primer | CARD8 | pLKO_check_R2 | GGATGAATACTGCCATTTGTCTCGAGG | reverse primer to amplify sequence in pLKO vector |
| gBlock | chimpanzee CARD8 | chimp_CARD8 | see NCBI accession XM_024351500.1 | gblock for chimpanzee CARD8 |
| sgRNA | CARD8 | CARD8_sgRNA | TTGTTAGCAAGGCGTCGCTGGGG | sgRNA for generating CARD8 KO |
| primer | chimpanzee CARD8 | cpz_CARD8_FF_internal_F | AAAACTGGAATTTTTTTTCAGGCTGAGGCCTG | Forward primer for Phe>Leu in chimp CARD8 |
| primer | chimpanzee CARD8 | cpz_CARD8_FF_internal_R | CAGGCCTCAGCCTGAAAAAAAATTCCAGTTTT | Reverse primer for Phe >Leu in chimp CARD8 |
| sgRNA | Caspase 1 | Casp1_sgRNA | GACAGTATTCCTAGAAGAACTGG | sgRNA for generating Casp1 KO |
| primer | Caspase 1 | Casp1_geno_F | ATTTATCCAATAATGGACAAGTCAAGCCG | forward primer for verifying CARD8 KO at sgRNA site |
| primer | Caspase 1 | Casp1_geno_R | CGAAGCAGTGAGATTTTTTTTCACATCTACG | reverse primer for verifying CARD8 KO at sgRNA site |

**Supplementary File 1b: List of Antibodies/Reagents**

| **Product** | **Catalog Number** | **Clone** | **Dilution Ratio** | **Purpose** | **Company** | **City** | **Country** | **State** |
| --- | --- | --- | --- | --- | --- | --- | --- | --- |
| mCherry mouse mAb | 632543 | **–** | 1:1000 | Western blot | Takara Bio | Kusatsu | Japan | **–** |
| p24 mouse mAb | ARP-3537 | **–** | 1:5000 | Western blot | NIH HIV Reagents Program | Manassas | USA | Virginia |
| vinculin mouse mAb | sc-73614 | 7F9 | 1:5000 | Western blot | Santa Cruz Biosciences | Santa Cruz | USA | California |
| CARD8 Rabbit pAb (C-terminal) | ab24186 | **–** | 1:1000 | Western blot | Abcam | Cambridge | United Kingdom | **–** |
| HIV-1 core antigen-FITC | 6604665 | KC57 | 1:300 | Flow cytometry | Beckman Coulter | Indianapolis | USA | Indiana |
| Pam3CSK4 | tlrl-pms | ­– | 500ng/mL | Priming | Invivogen | San Diego | USA | California |
| CL075 (3M002) | tlrl-c75 | – | 6ng/mL | Priming | Invivogen | San Diego | USA | California |
| TL8-506 | tlrl-tl8506 | – | 25ng/mL | Priming | Invivogen | San Diego | USA | California |
| LPS-EB Ultrapure, 5 x 106 EU | tlrl-3pelps | – | 5µg/mL | Priming | Invivogen | San Diego | USA | California |
| FLICA 660 Caspase-1 Kit | 9122 | – | 1:60-1:100 | Flow cytometry | Immunocytochemistry Technologies | Davis | USA | California |
| Val-boro-Pro | 531465 | ­– | 10µM | Drug | Millipore Sigma | Burlington | USA | Massachusetts |
| Nigericin | tlrl-nig | – | 5µg/mL | Drug | Invivogen | San Diego | USA | California |
| Lopinavir | HRP-9481 | ­– | 5µM | Drug | NIH HIV Reagents Program | Manassas | USA | Virginia |

**Supplementary File 1c: Primate CARD8 gene IDs**

| **Species** | **NCBI ID** | **Ensembl ID** |
| --- | --- | --- |
| Homo sapiens (human) | NM_001351782.2 |  |
| Homo neanderthalensis (neanderthal) |  | ENST00000357778 + ENST00000391898 |
| Pan troglodytes (chimpanzee) | XM_024351500.1 |  |
| Pan paniscus (bonobo) | XM_003814099.5 |  |
| Gorilla gorilla (gorilla) |  | ENSGGOT00000000261.3 |
| Pongo abelii (Sumatran orangutan) |  | ENSPPYT00000036267.1 |
| Nomascus leucogenys (Northern white-cheeked gibbon) | XM_030820964.1 |  |
| Hylobates moloch (silvery gibbon) | XM_032169303.1 |  |
| Macaca thibetana (Tibetan macaque) | XR_007721767.1 |  |
| Macaca fascicularis (Crab-eating macaque) | XM_015441314.1 |  |
| Macaca mulatta (rhesus macaque | XR_003724816.1 |  |
| Cercocebus atys (sooty mangabey) |  | ENSCATT00000020868.1 |
| Rhinopithecus roxellana (golden snub-nosed monkey) | XM_030942634.1 |  |
| Trachypithecus francoisi (Francois' leaf monkey) | XM_033223854.1 |  |
| Cebus imitator (Panamanian White-faced Capuchin) | XM_017500443.2 |  |
| Cebus apella (Tufted capuchin) | XM_032271013.1 |  |
| Saimiri boliviensis (Black-capped squirrel monkey) | XM_003940456.3 |  |
| Aotus nancymaae (Nancy Ma's night monkey) | XM_021668509.1 |  |
| Callithrix jacchus (common marmoset) | XM_008988319.4 |  |
