# Supplementary figures and images for: A human-specific motif facilitates CARD8 inflammasome activation after HIV-1 infection
Source: eLife. 2023 Jul 7;12:e84108. doi: 10.7554/eLife.84108 (PMC10359095; doi:10.7554/eLife.84108)

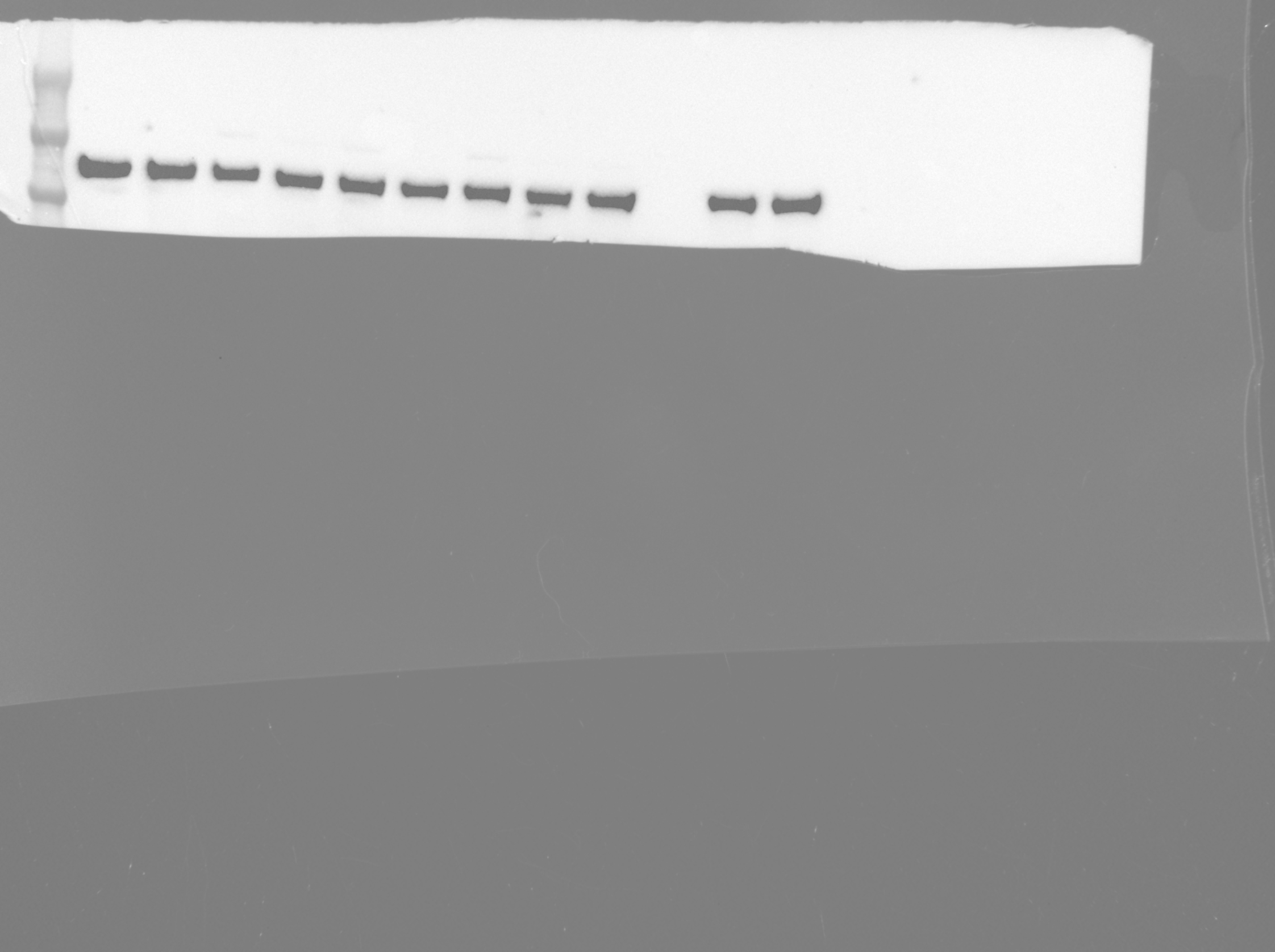

Supplement: Source data 1. [file elife-84108-data1.zip › WesternBlot_SourceData_tifs/Figure2A_SourceData_vinculin.tif]

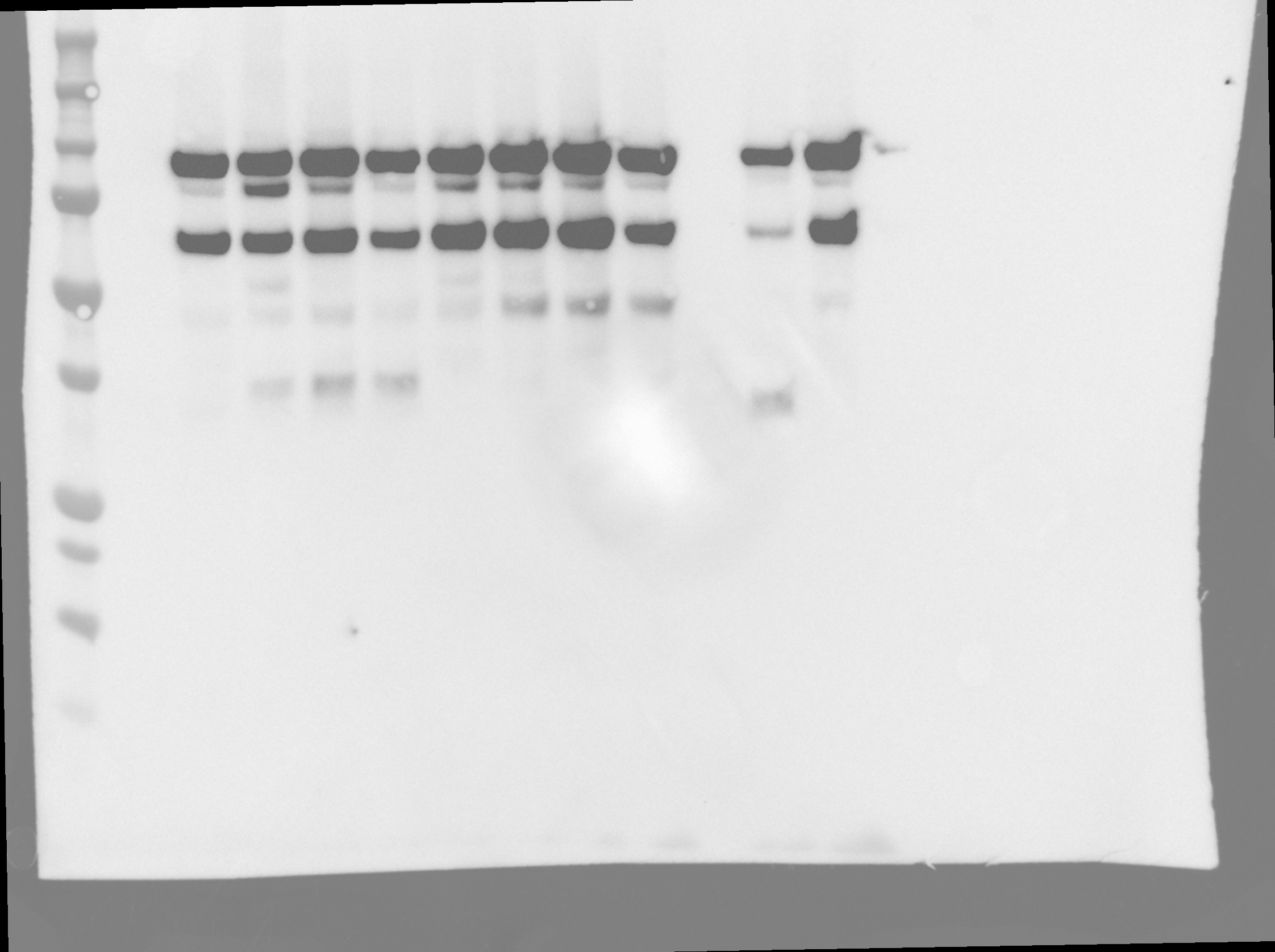

Supplement: Source data 1. [file elife-84108-data1.zip › WesternBlot_SourceData_tifs/Figure2A_SourceData_mCherry.tif]

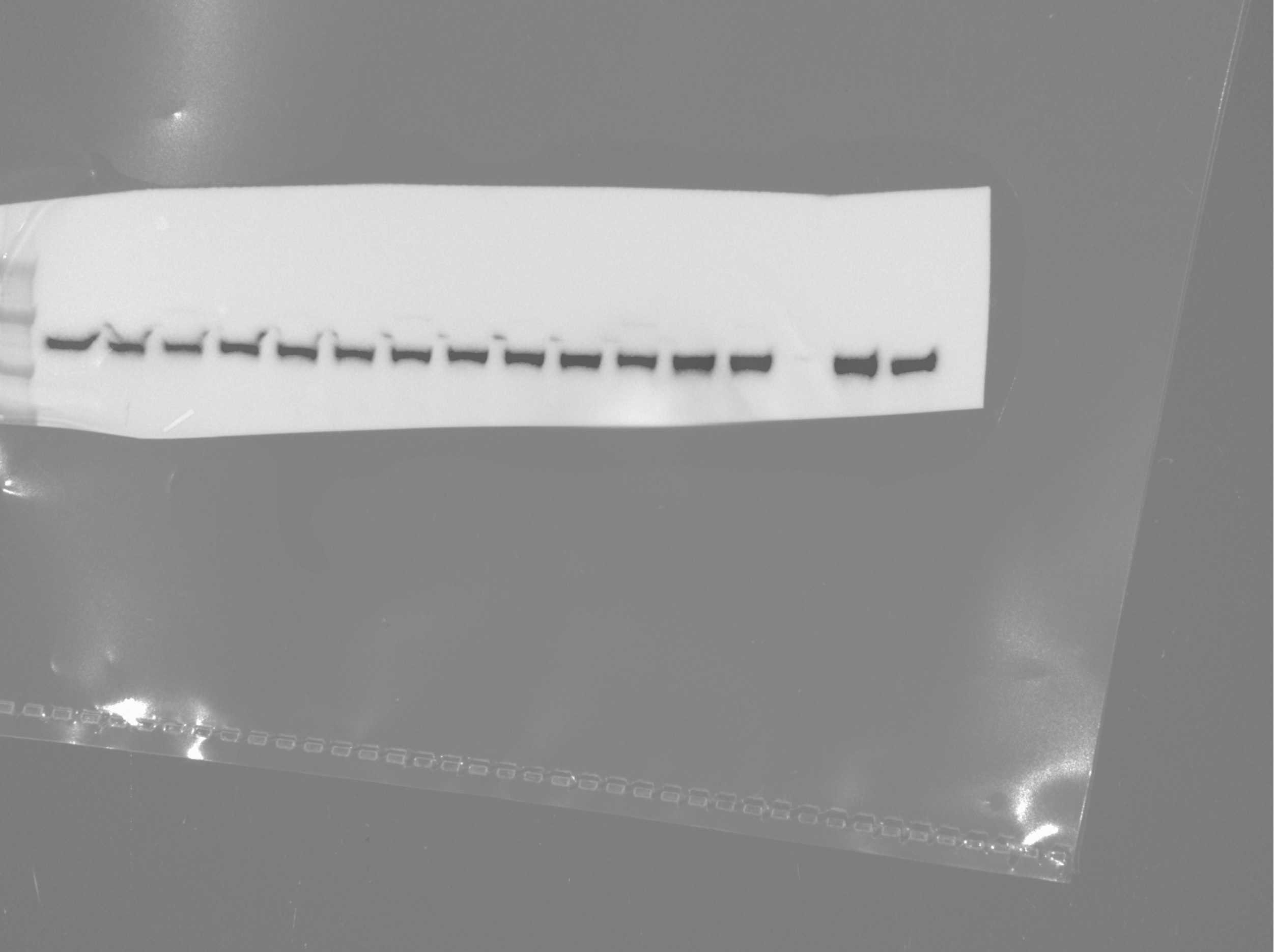

Supplement: Source data 1. [file elife-84108-data1.zip › WesternBlot_SourceData_tifs/Figure1D_SourceData_vinculin.tif]

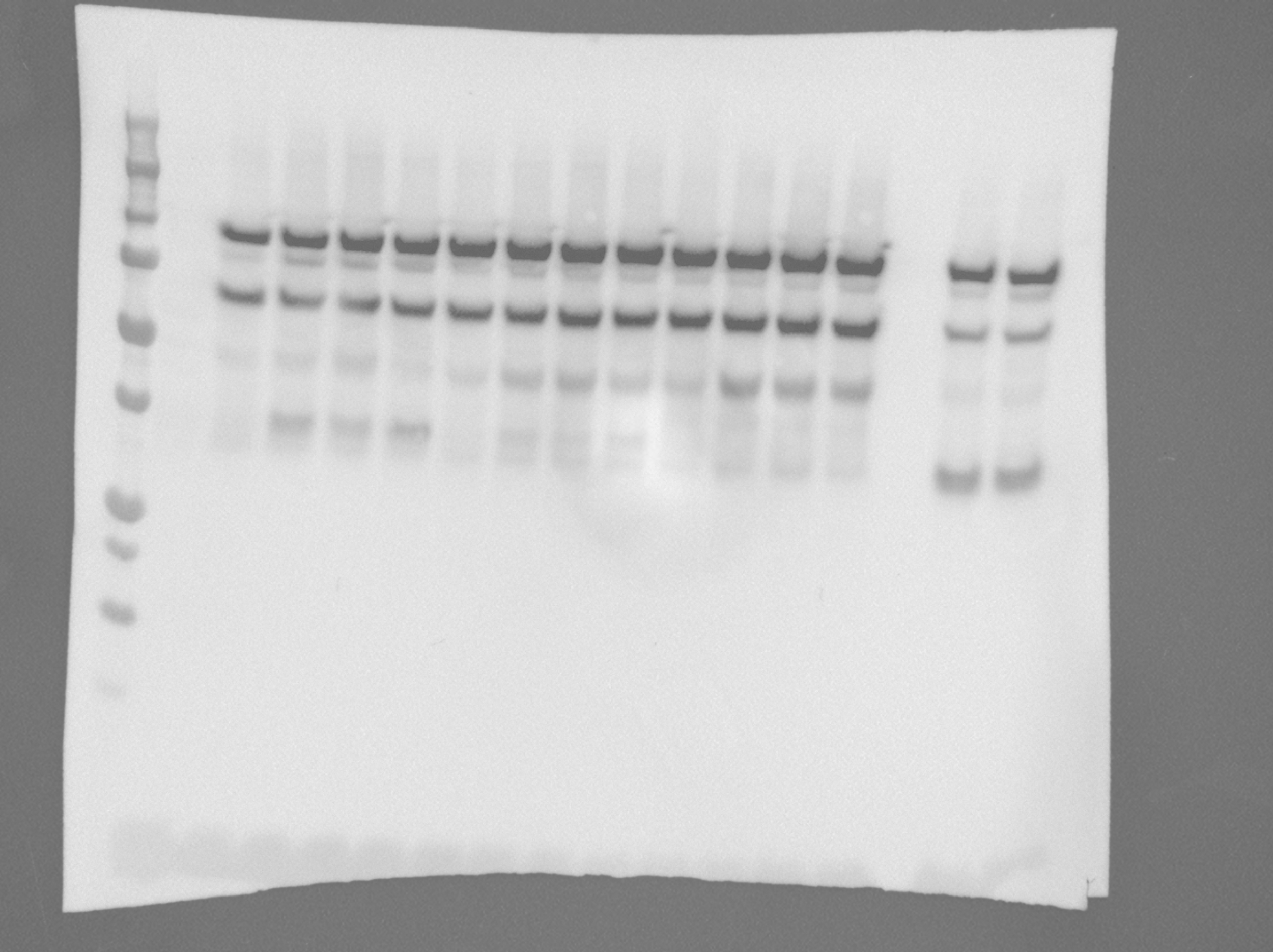

Supplement: Source data 1. [file elife-84108-data1.zip › WesternBlot_SourceData_tifs/Figure1D_SourceData_mCherry.tif]

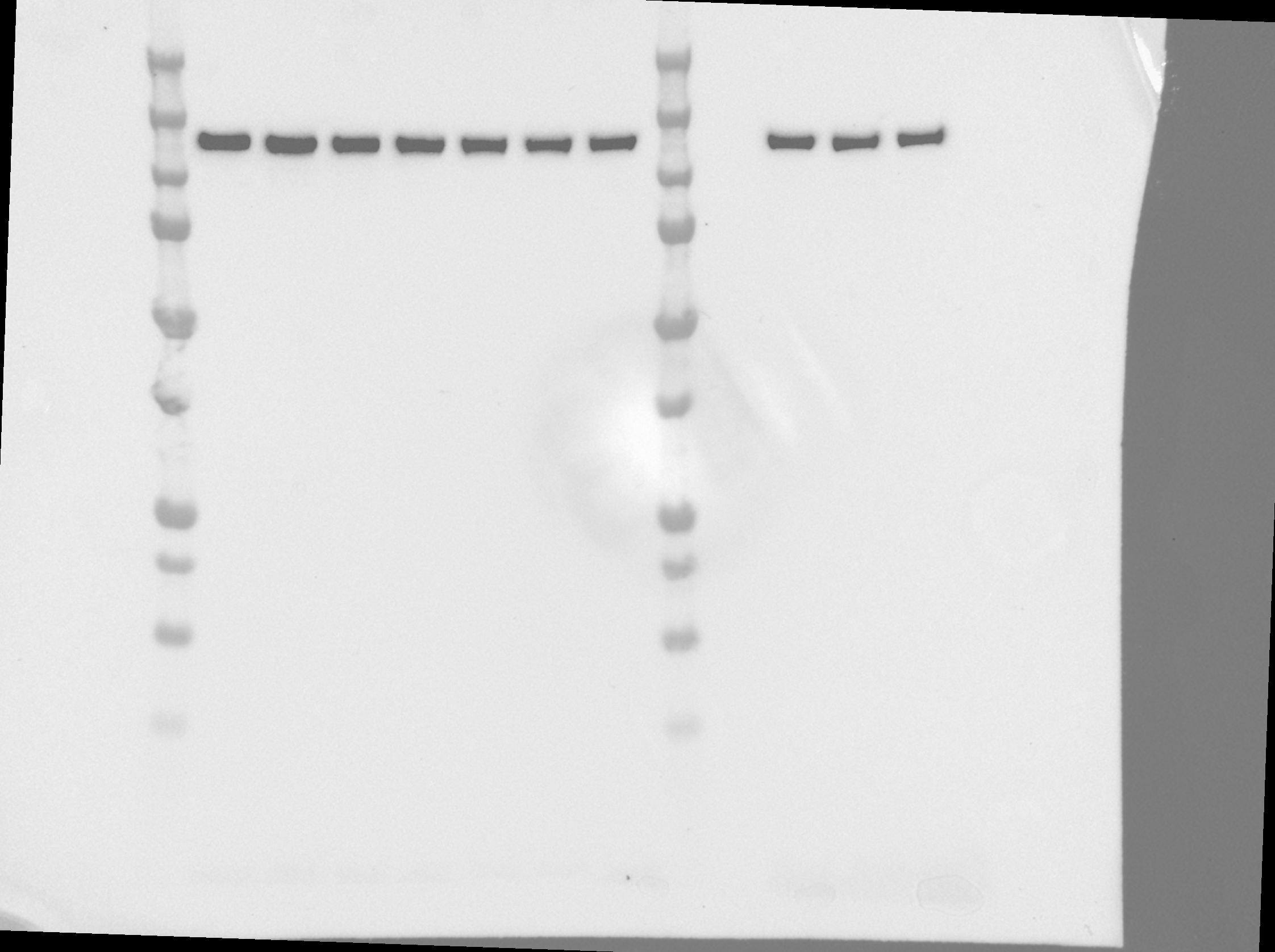

Supplement: Source data 1. [file elife-84108-data1.zip › WesternBlot_SourceData_tifs/Figure3-Supp1A_SourceData_vinculin.tif]

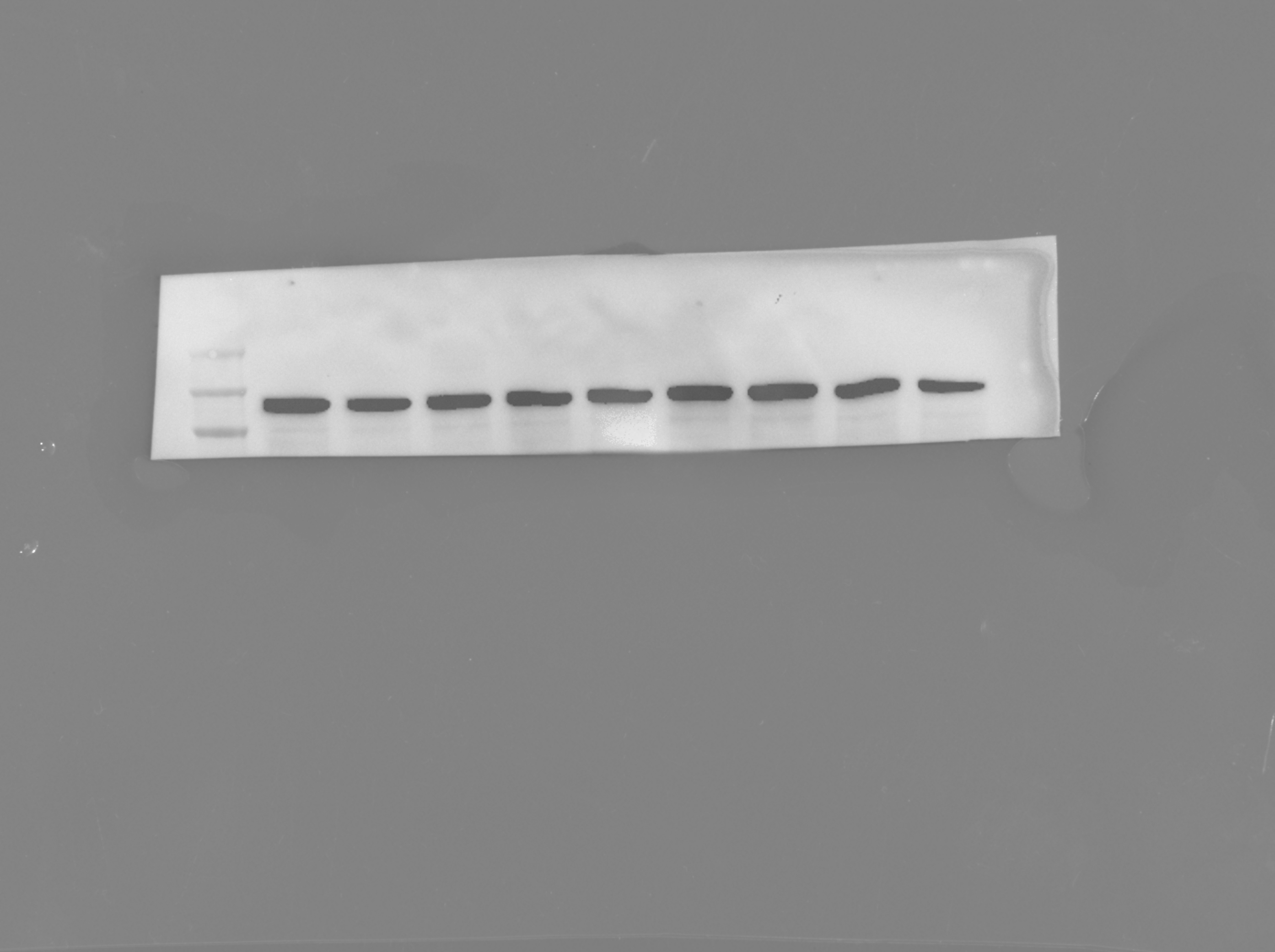

Supplement: Source data 1. [file elife-84108-data1.zip › WesternBlot_SourceData_tifs/Figure5D_SourceData_vinculin.tif]

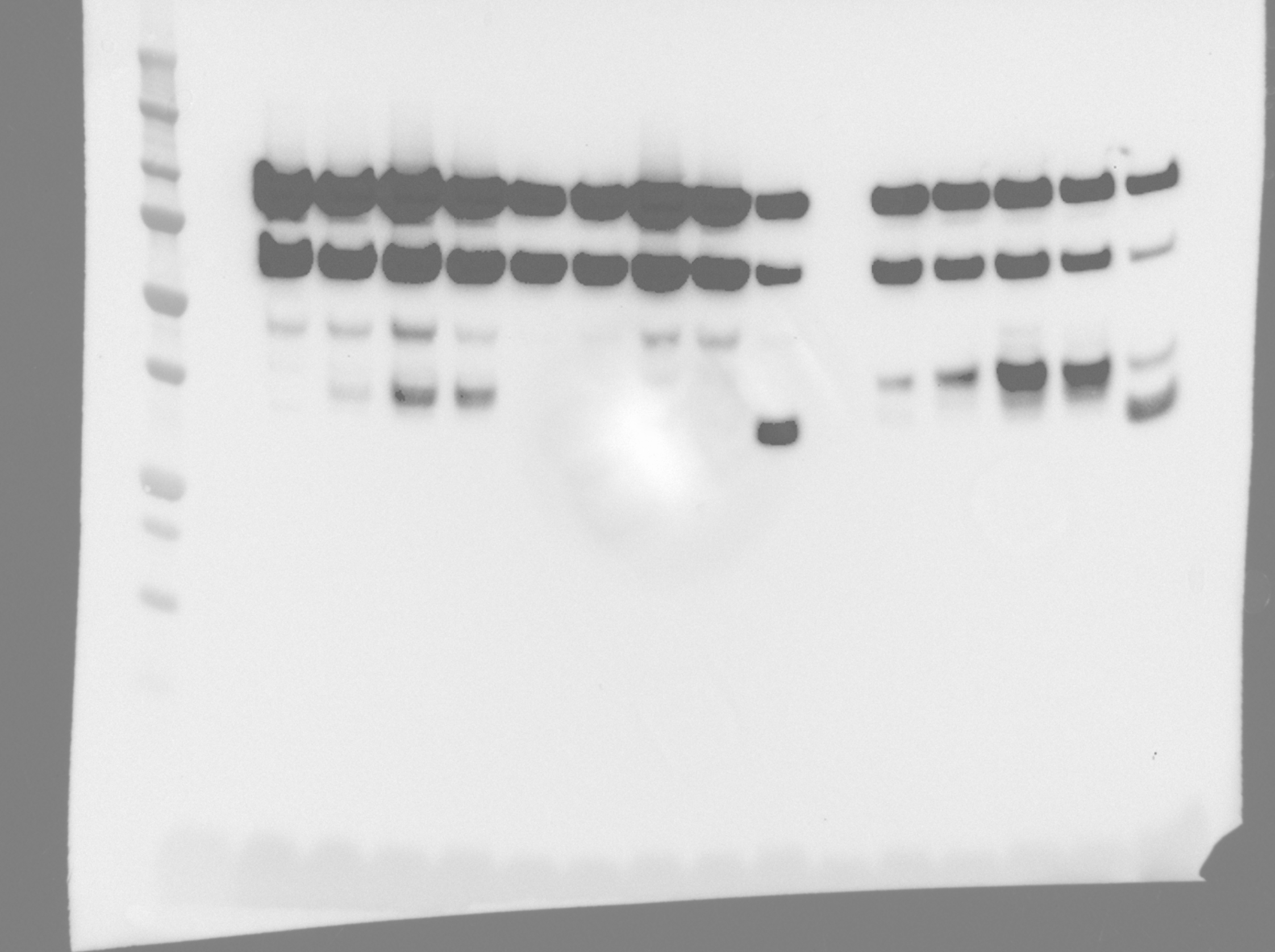

Supplement: Source data 1. [file elife-84108-data1.zip › WesternBlot_SourceData_tifs/Figure1C_SourceData_mCherry.tif]

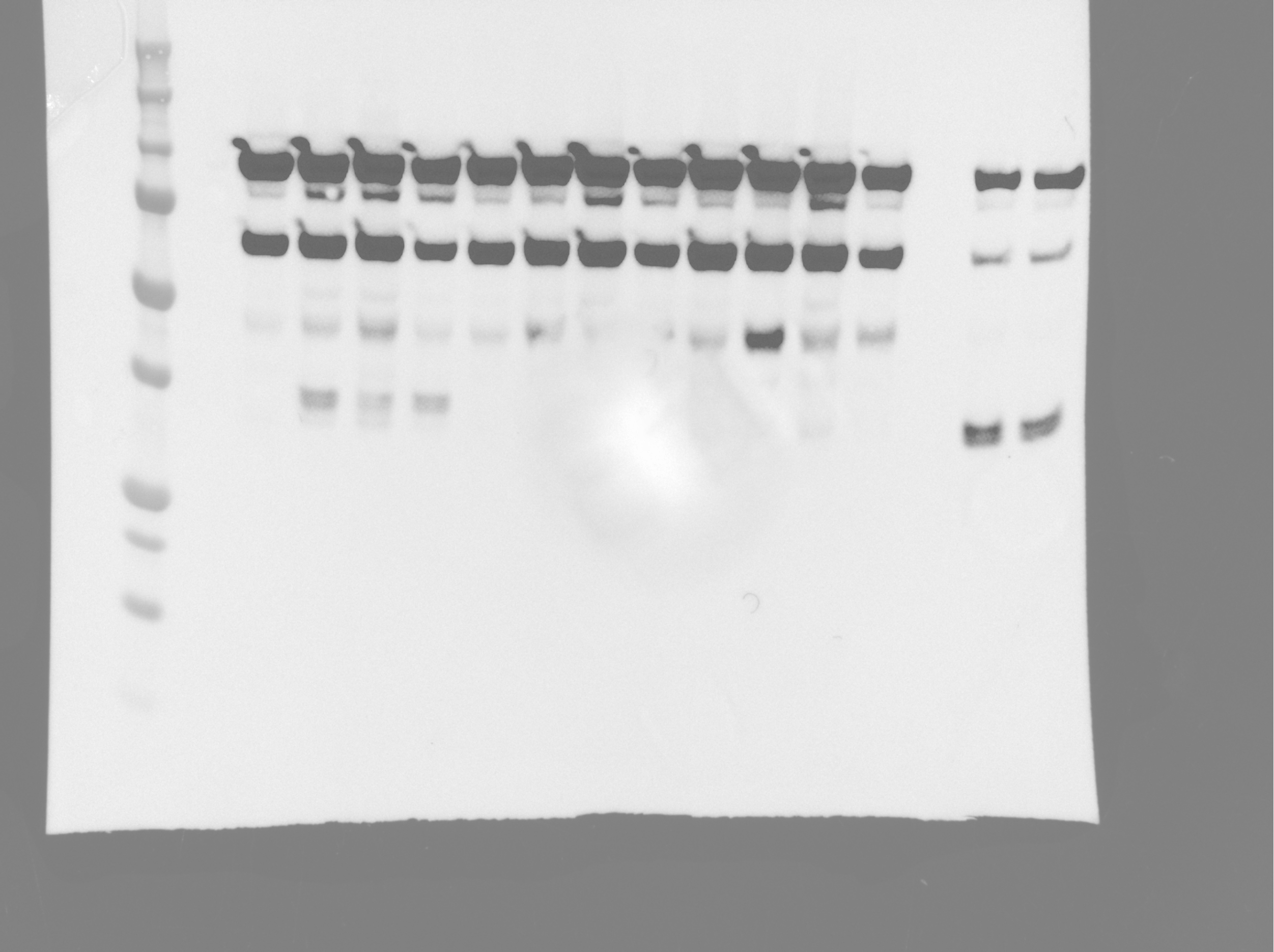

Supplement: Source data 1. [file elife-84108-data1.zip › WesternBlot_SourceData_tifs/Figure2B_SourceData_mCherry.tif]

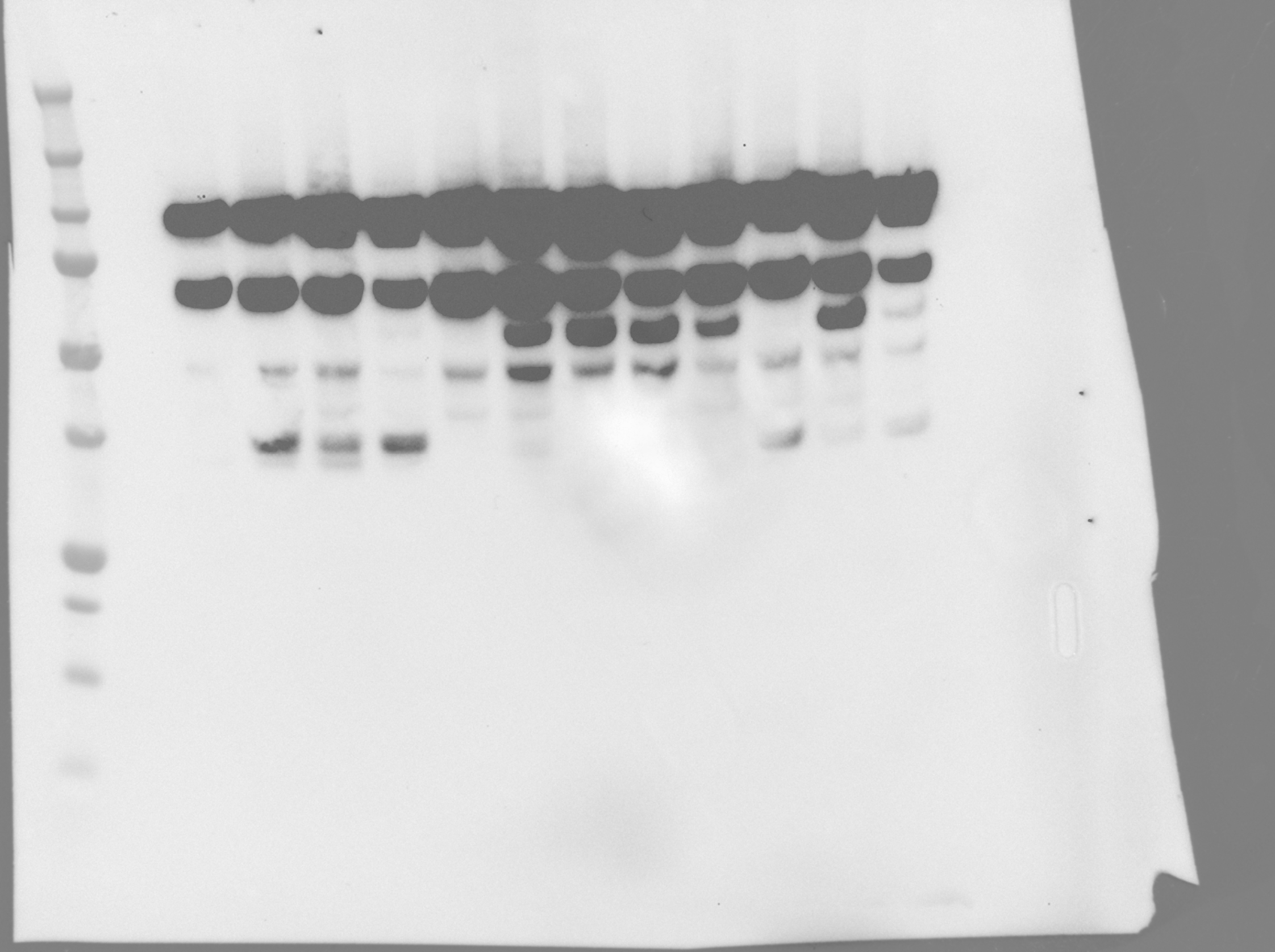

Supplement: Source data 1. [file elife-84108-data1.zip › WesternBlot_SourceData_tifs/Figure5-Supp1_SourceData_mCherry.tif]

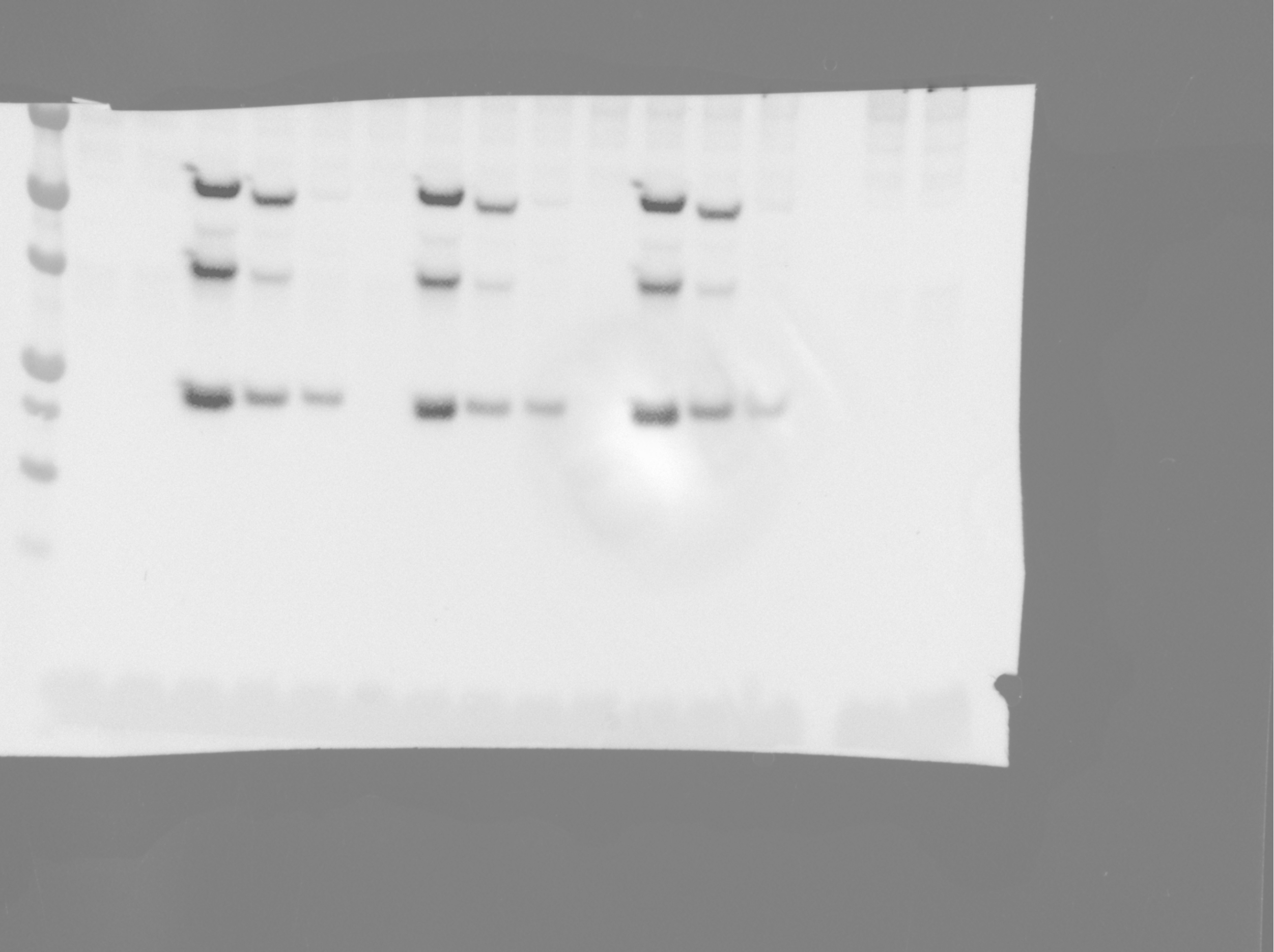

Supplement: Source data 1. [file elife-84108-data1.zip › WesternBlot_SourceData_tifs/Figure1D_Source Data_p24.tif]

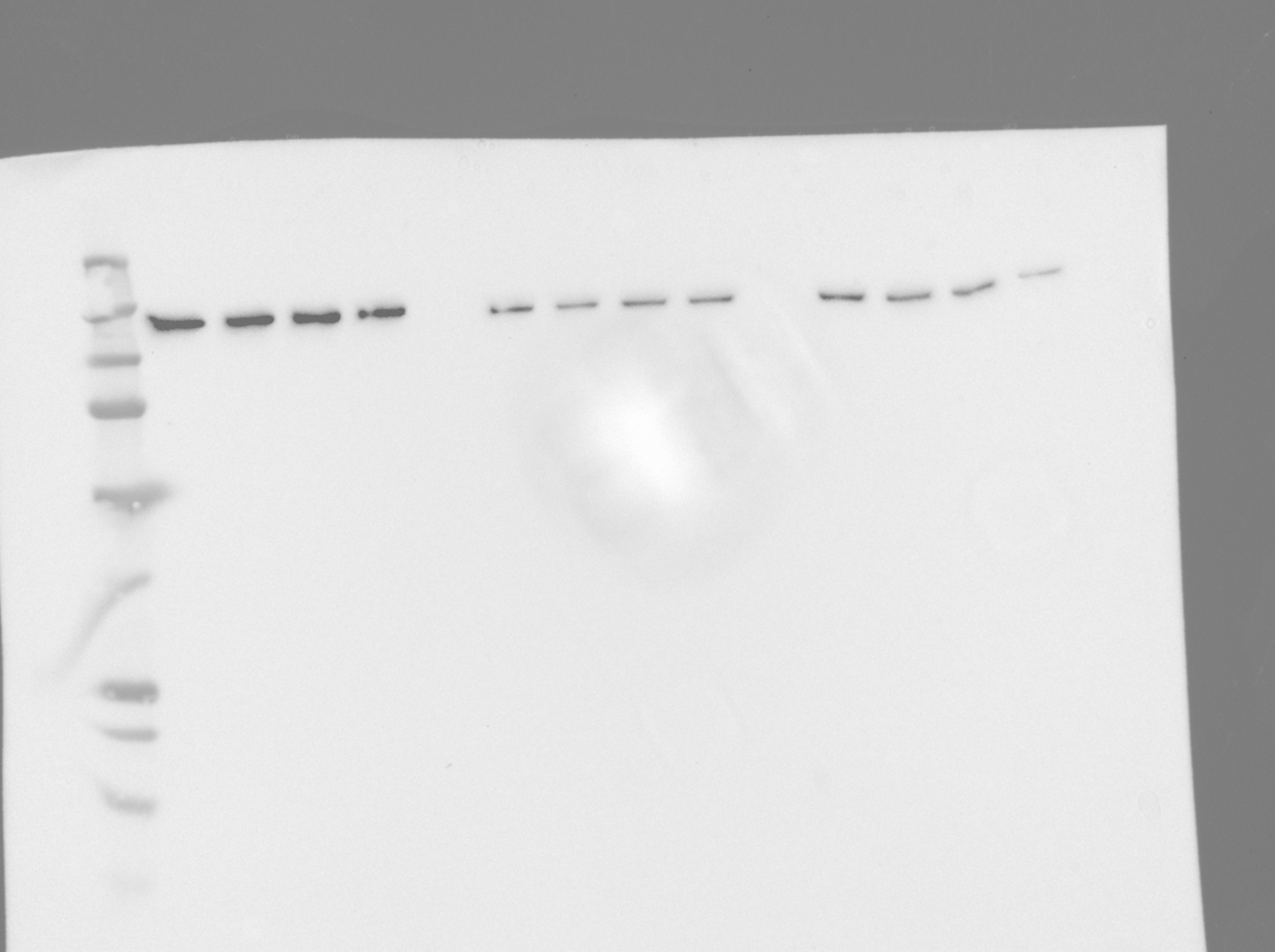

Supplement: Source data 1. [file elife-84108-data1.zip › WesternBlot_SourceData_tifs/Figure3A_SourceData_vinculin.tif]

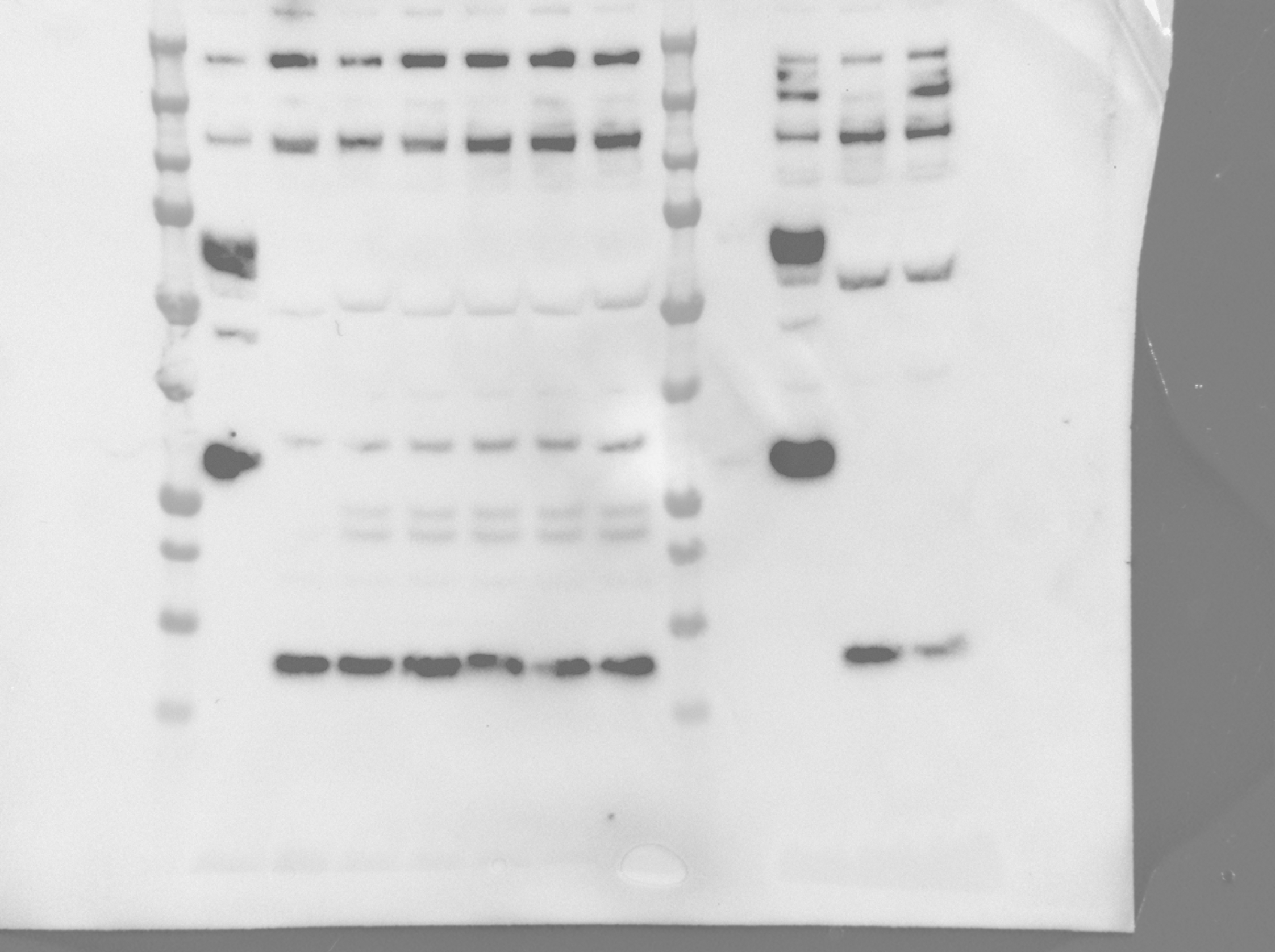

Supplement: Source data 1. [file elife-84108-data1.zip › WesternBlot_SourceData_tifs/Figure3-Supp1A_SourceData_CARD8-C.tif]

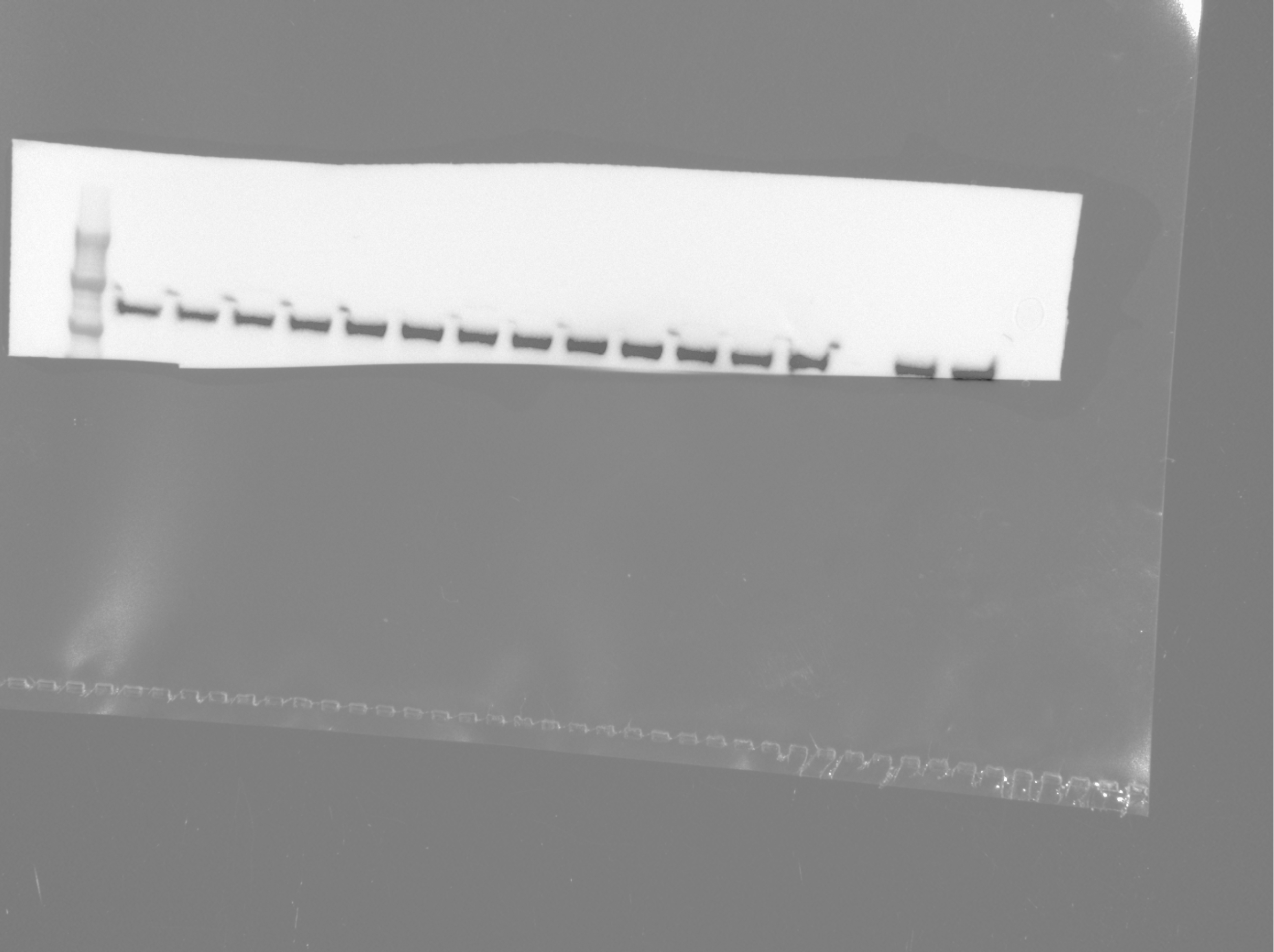

Supplement: Source data 1. [file elife-84108-data1.zip › WesternBlot_SourceData_tifs/Figure2B_SourceData_vinculin.tif]

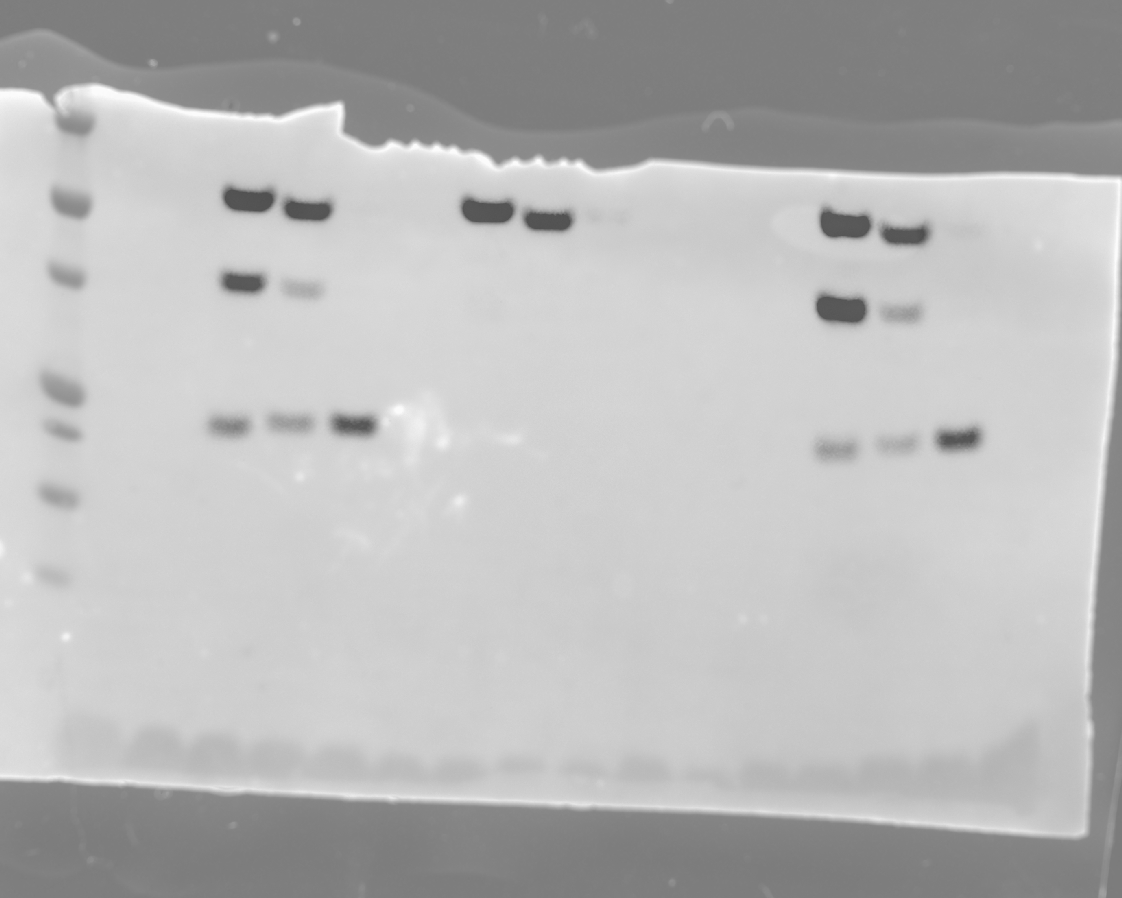

Supplement: Source data 1. [file elife-84108-data1.zip › WesternBlot_SourceData_tifs/Figure1C_SourceData_p24.tif]

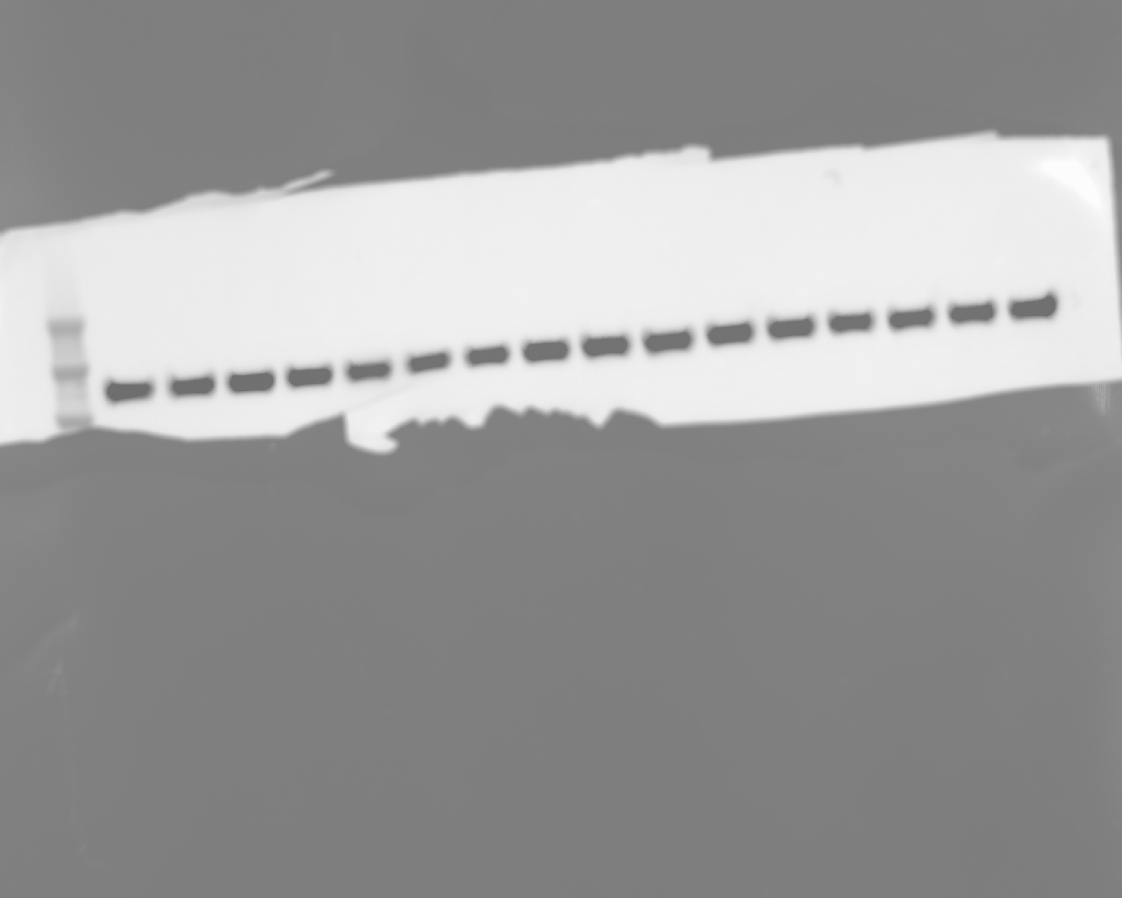

Supplement: Source data 1. [file elife-84108-data1.zip › WesternBlot_SourceData_tifs/Figure1C_SourceData_vinculin.tif]

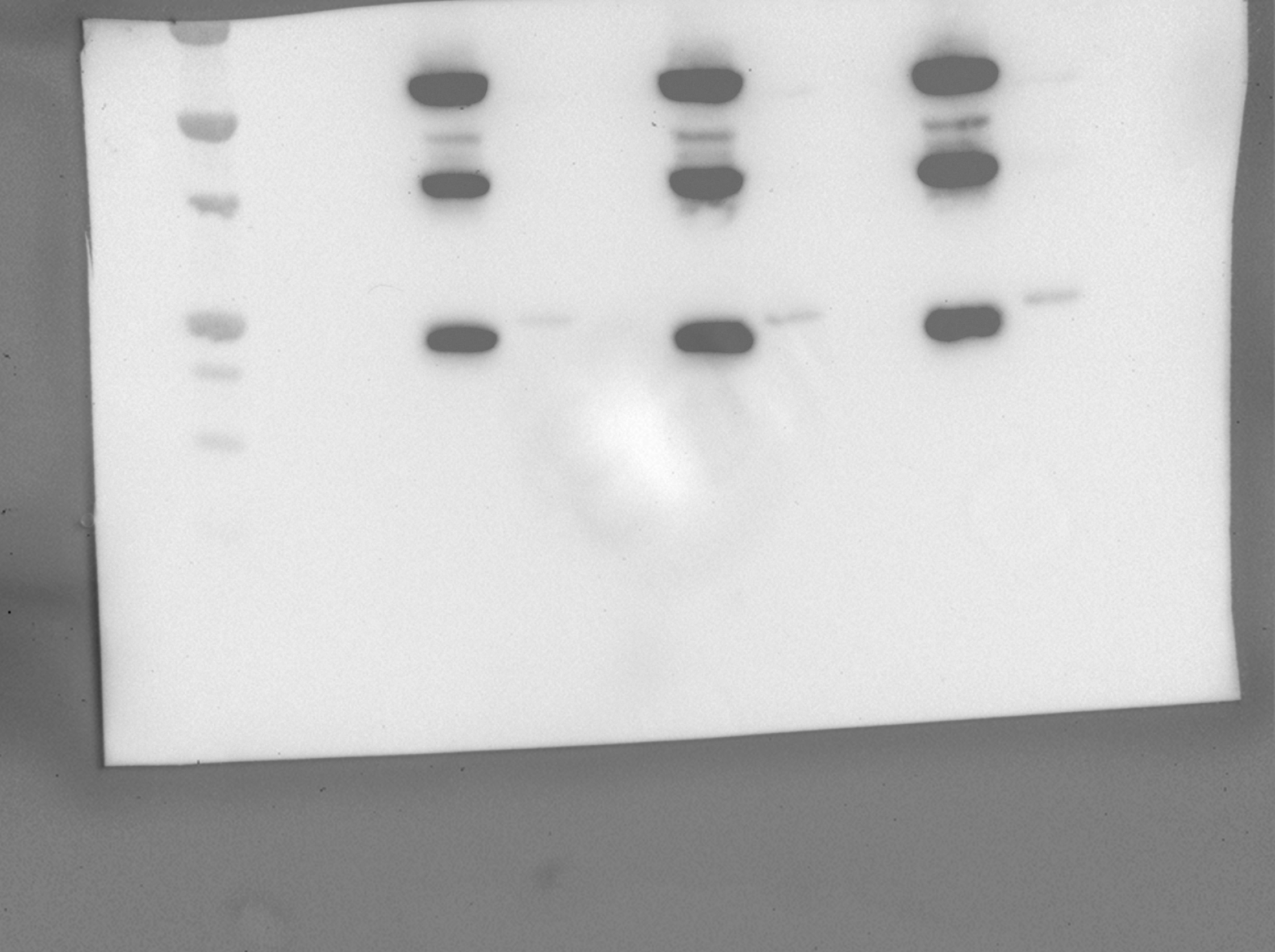

Supplement: Source data 1. [file elife-84108-data1.zip › WesternBlot_SourceData_tifs/Figure2-Supp1_SourceData_p24.tif]

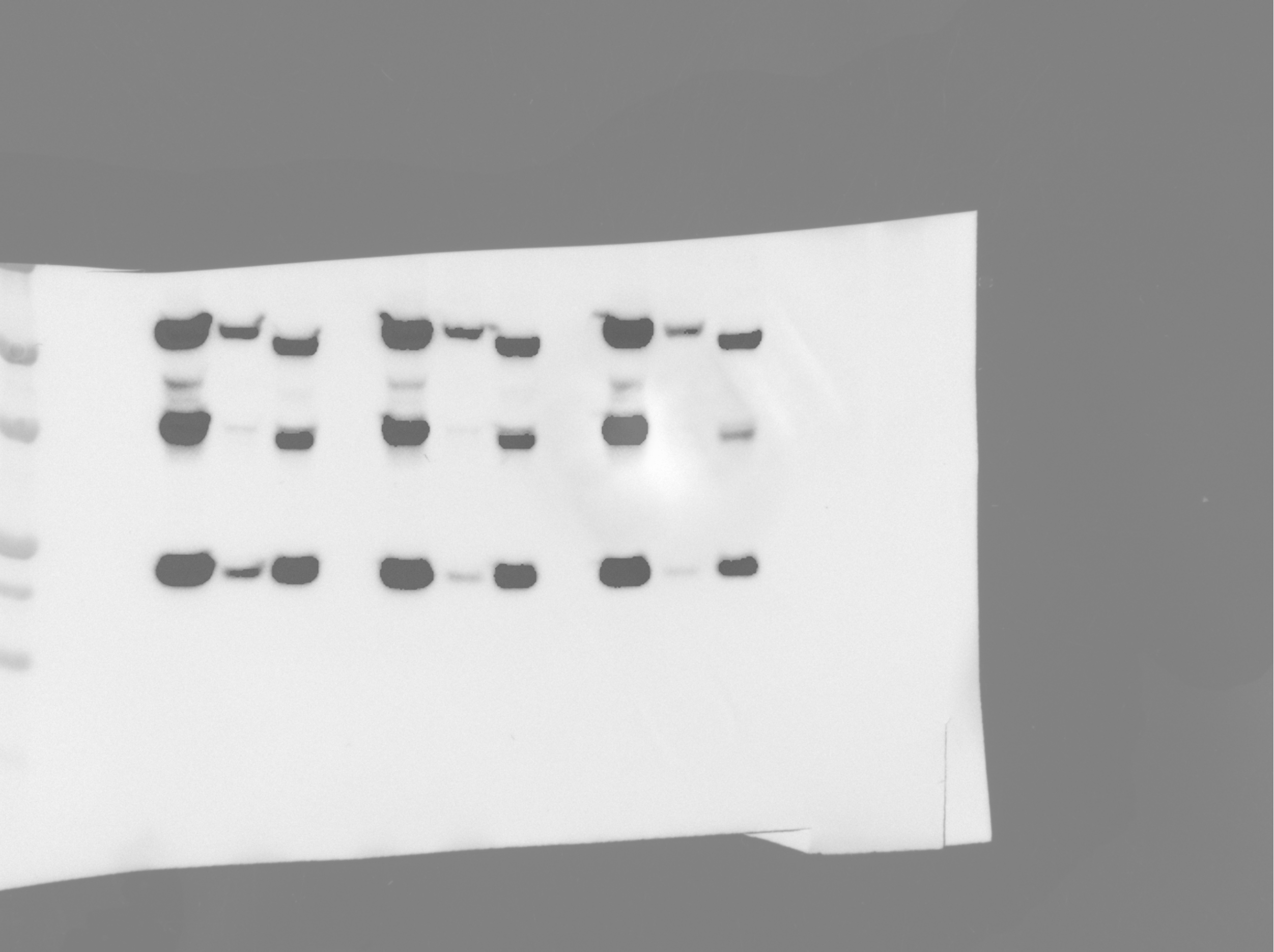

Supplement: Source data 1. [file elife-84108-data1.zip › WesternBlot_SourceData_tifs/Figure2B_SourceData_p24.tif]

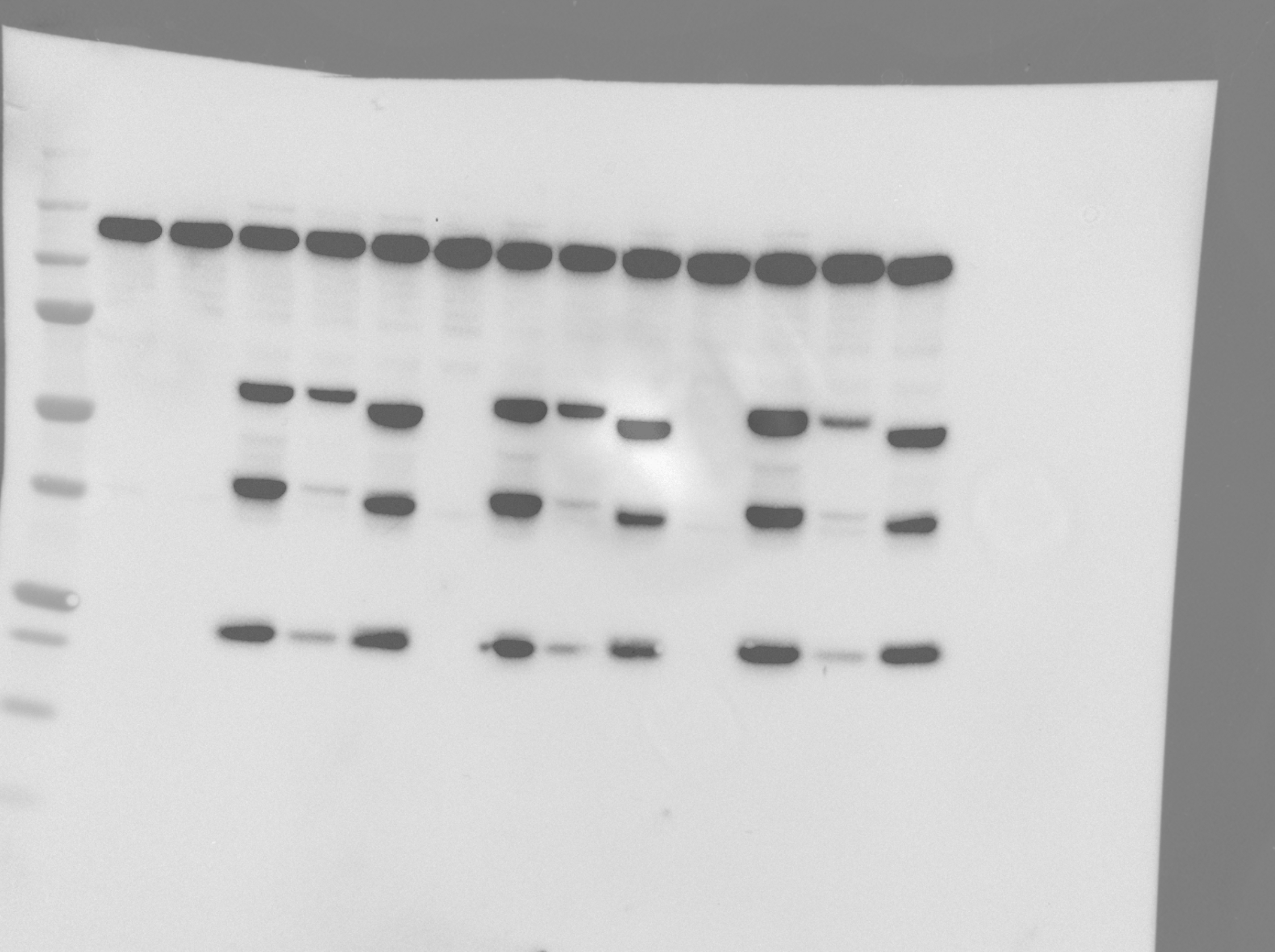

Supplement: Source data 1. [file elife-84108-data1.zip › WesternBlot_SourceData_tifs/Figure5-Supp1_SourceData_p24_vinculin.tif]

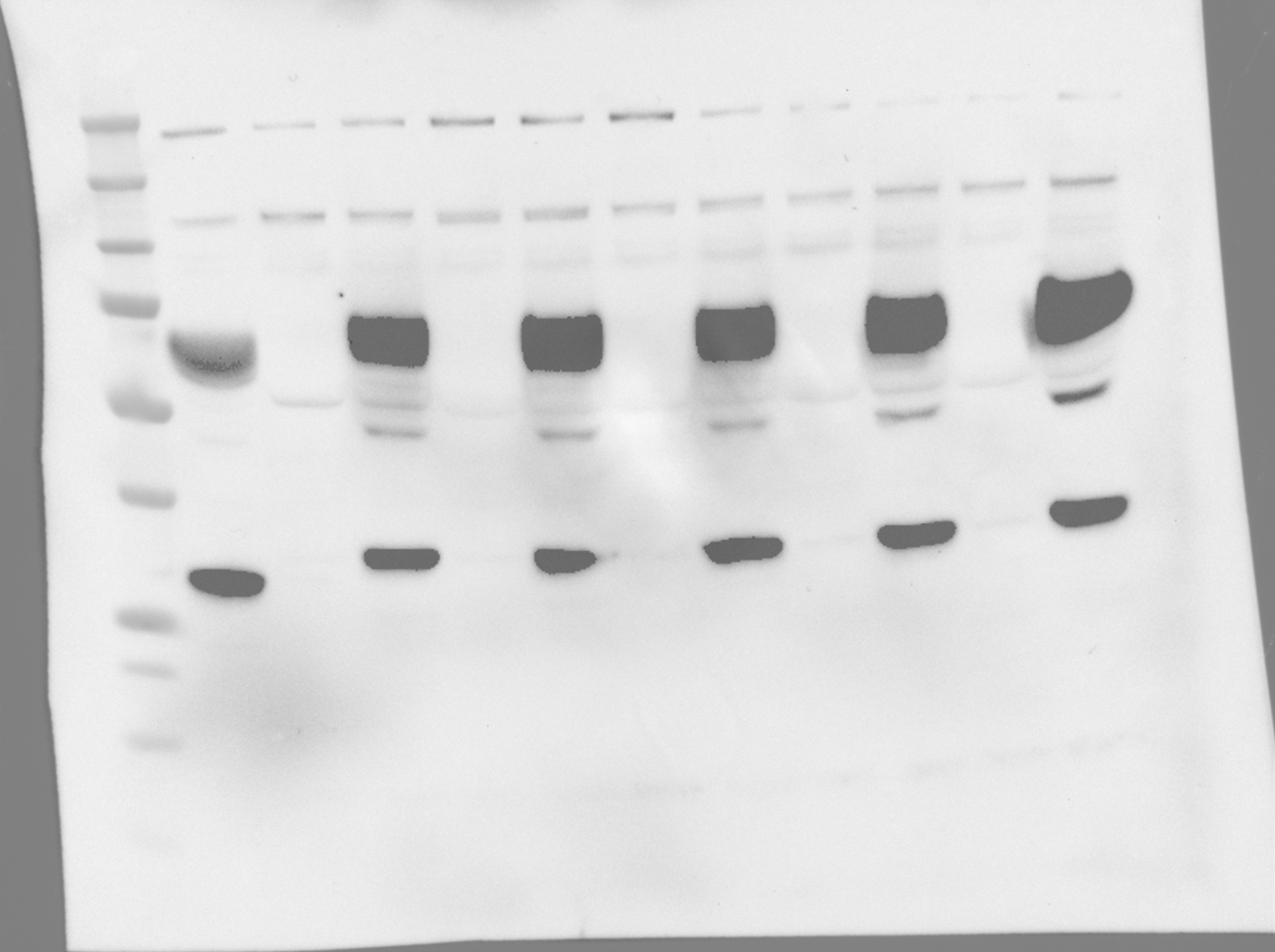

Supplement: Source data 1. [file elife-84108-data1.zip › WesternBlot_SourceData_tifs/Figure5A_SourceData_CARD8-C.tif]

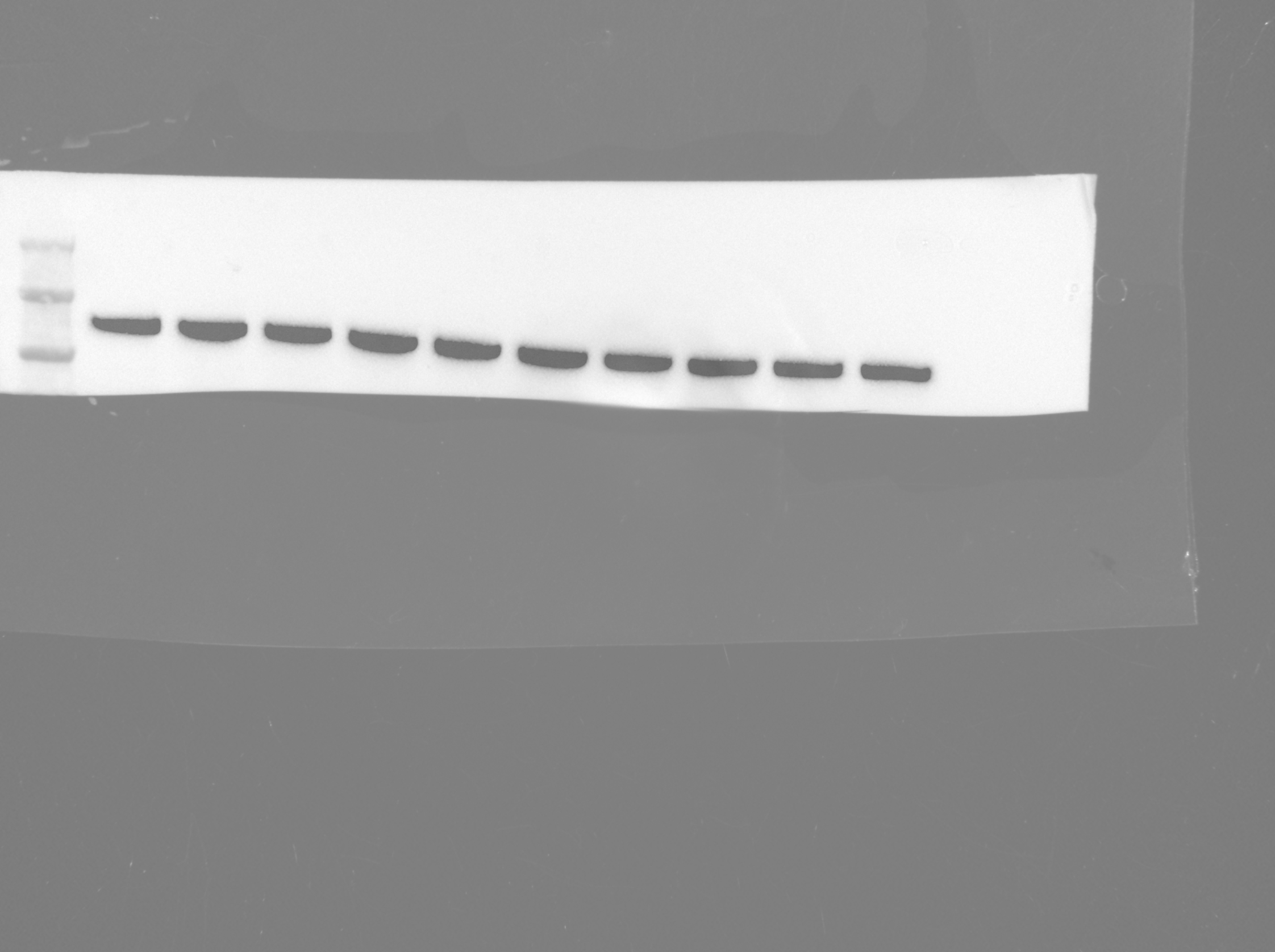

Supplement: Source data 1. [file elife-84108-data1.zip › WesternBlot_SourceData_tifs/Figure2-Supp1_SourceData_vinculin.tif]

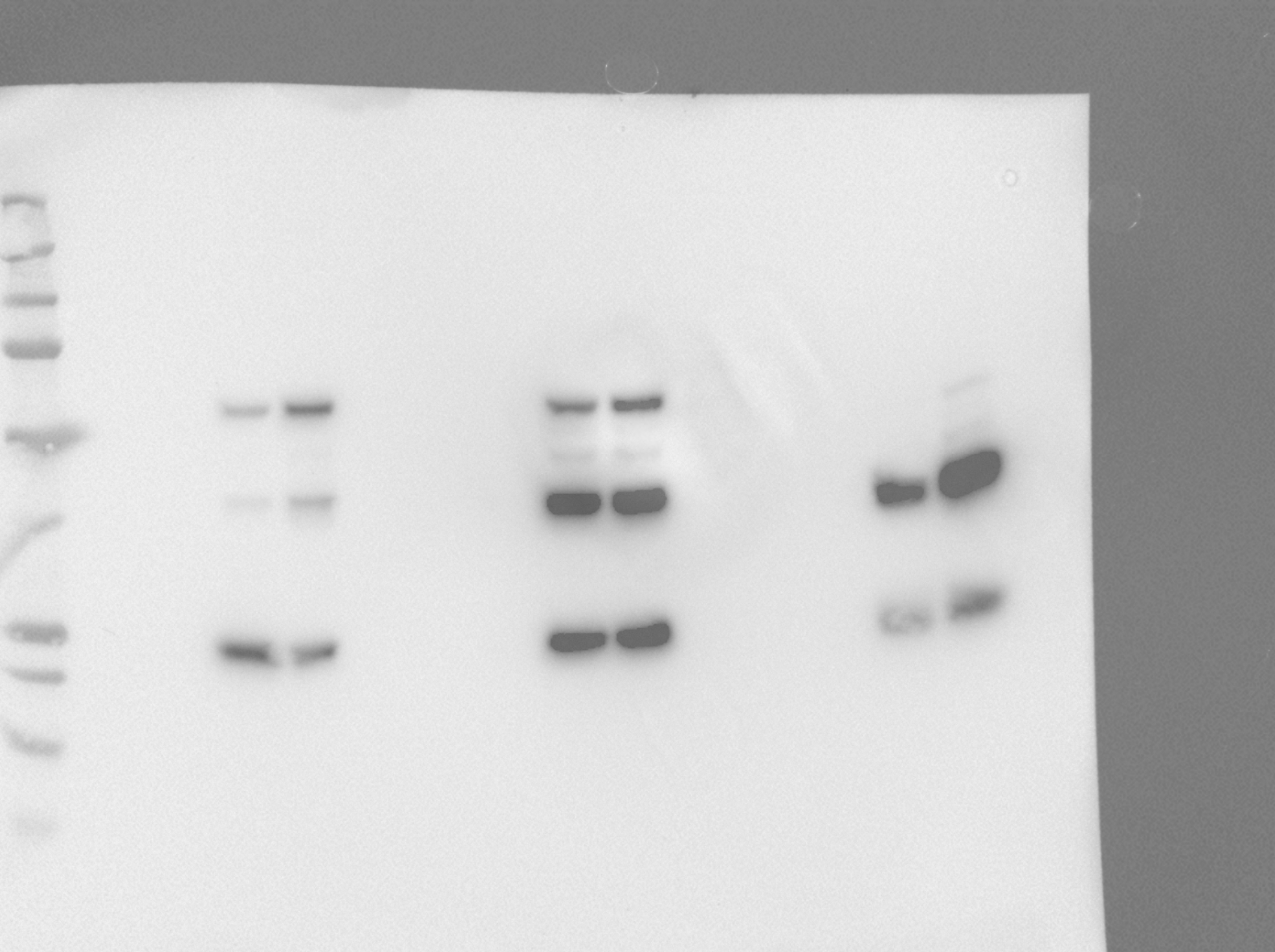

Supplement: Source data 1. [file elife-84108-data1.zip › WesternBlot_SourceData_tifs/Figure3A_SourceData_p24.tif]

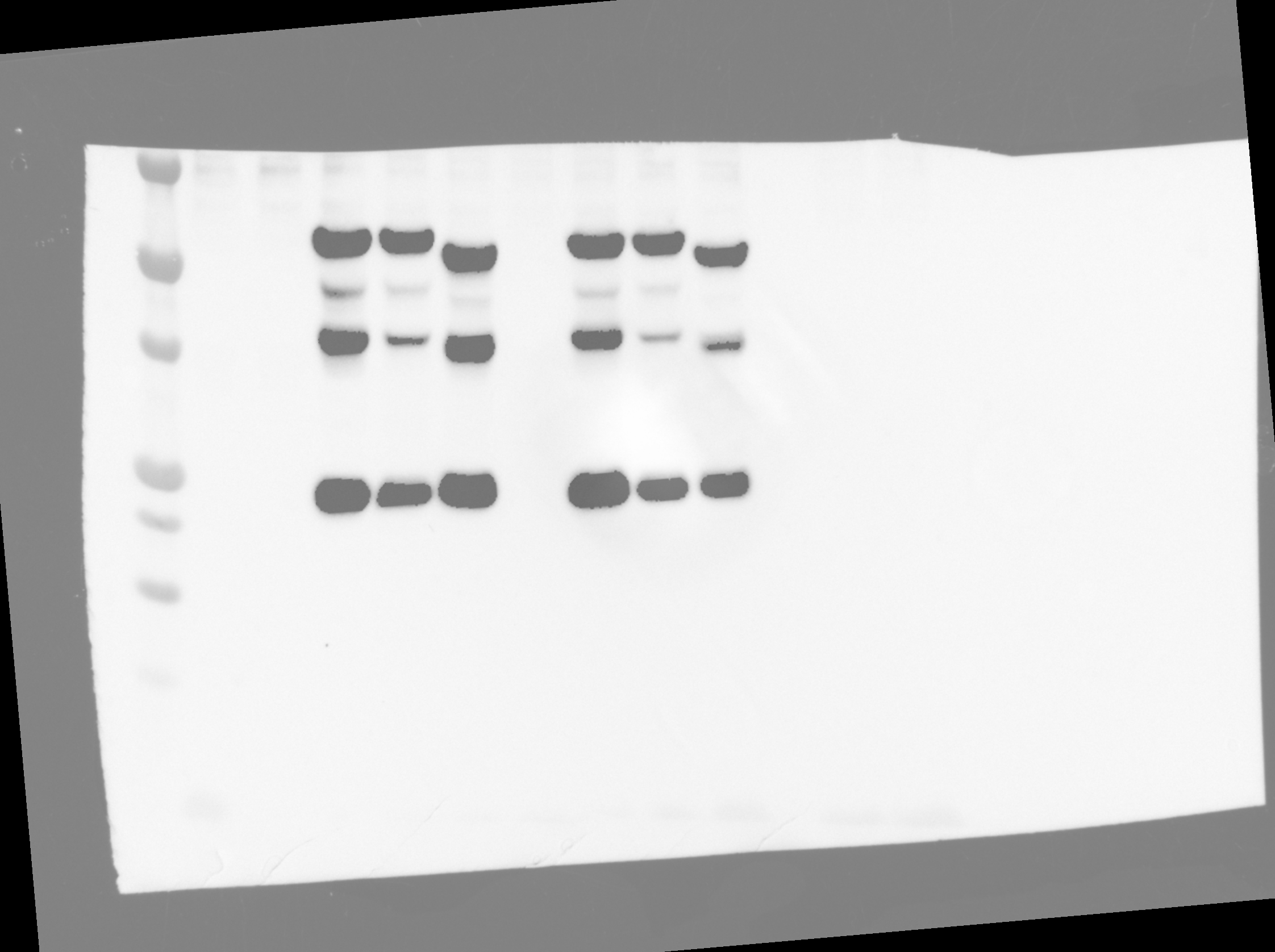

Supplement: Source data 1. [file elife-84108-data1.zip › WesternBlot_SourceData_tifs/Figure2A_SourceData_p24.tif]

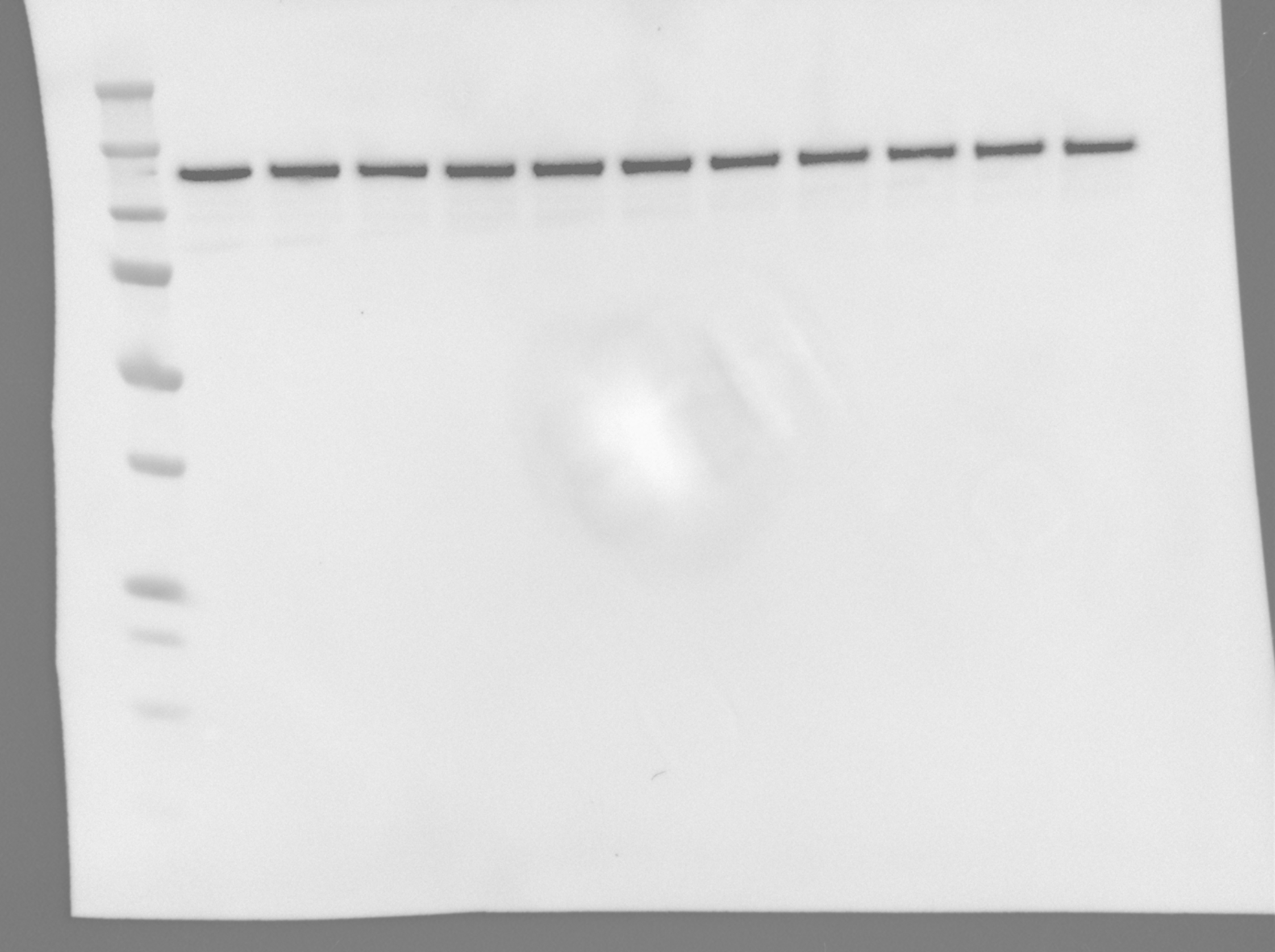

Supplement: Source data 1. [file elife-84108-data1.zip › WesternBlot_SourceData_tifs/Figure5A_SourceData_vinculin.tif]

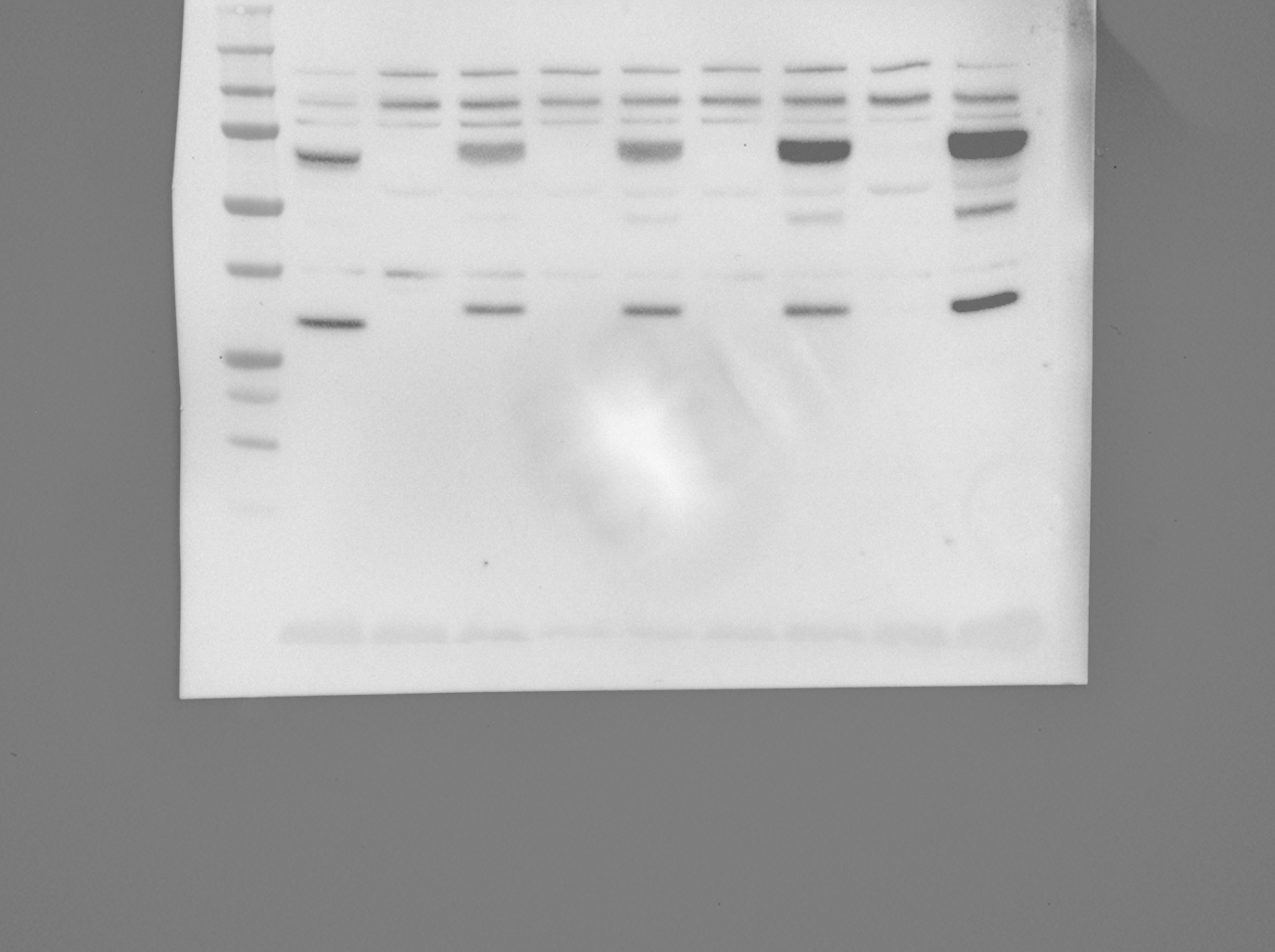

Supplement: Source data 1. [file elife-84108-data1.zip › WesternBlot_SourceData_tifs/Figure5D_SourceData_CARD8-C.tif]

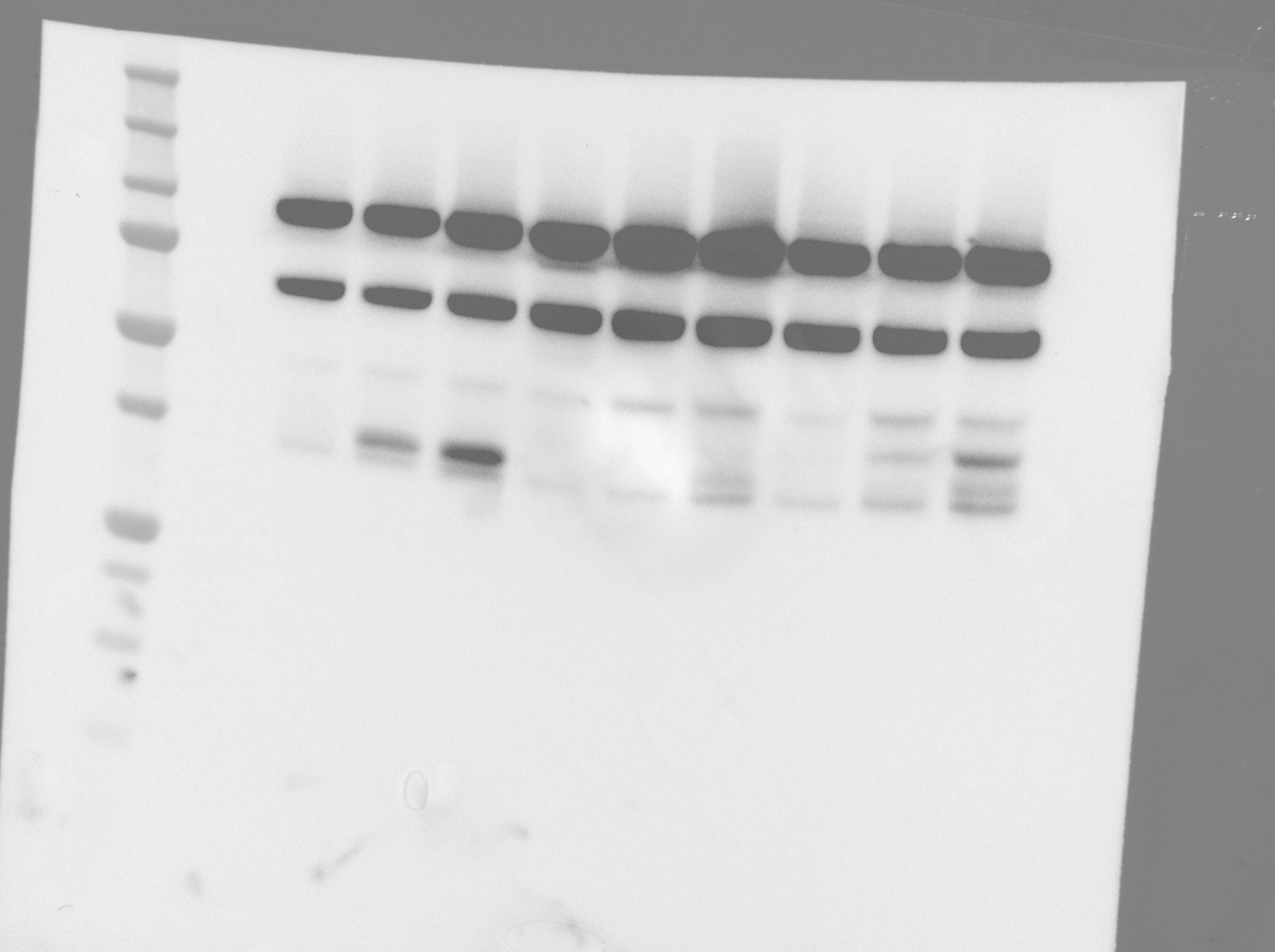

Supplement: Source data 1. [file elife-84108-data1.zip › WesternBlot_SourceData_tifs/Figure2-Supp1_SourceData_mCherry.tif]
