# Supplementary material for: A human-specific motif facilitates CARD8 inflammasome activation after HIV-1 infection
Source: eLife. 2023 Jul 7;12:e84108. doi: 10.7554/eLife.84108 (PMC10359095; doi:10.7554/eLife.84108)
Supplement: Source data 2. [file elife-84108-data2.pdf]

Figure 1C

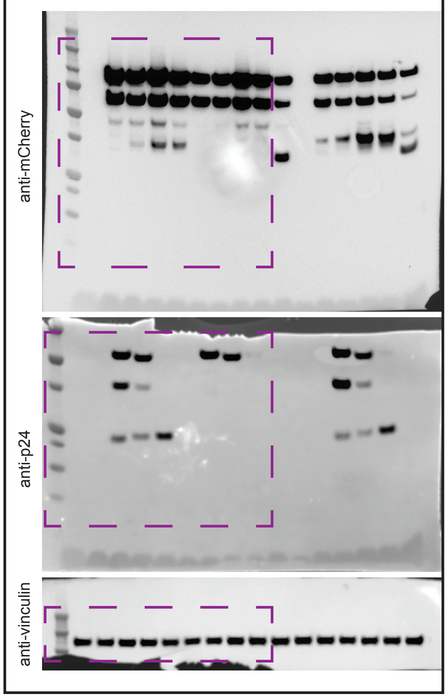

Figure 1D

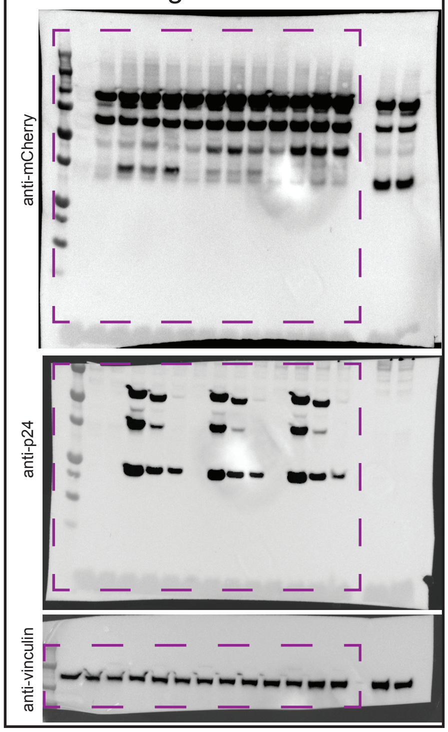

Figure 2A

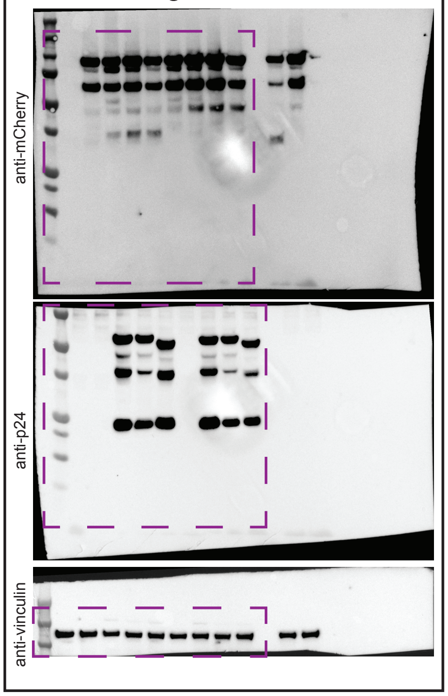

Figure 2B

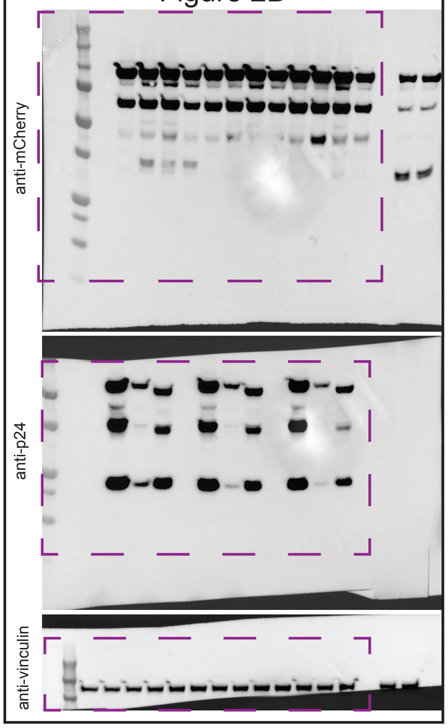

Figure 3A

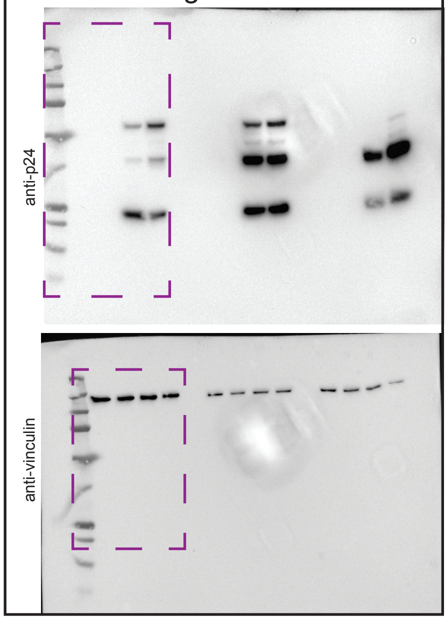

Figure 5A

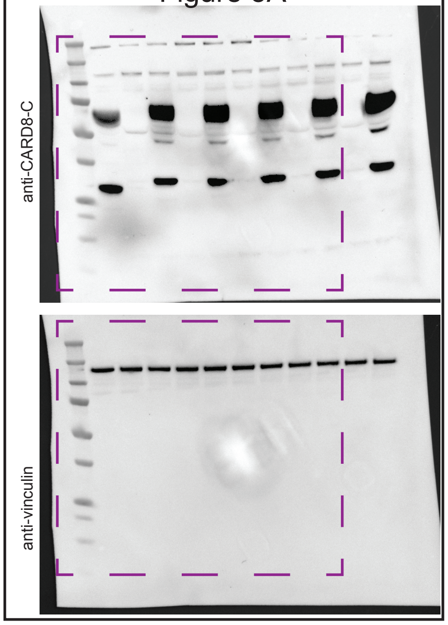

Figure 5D

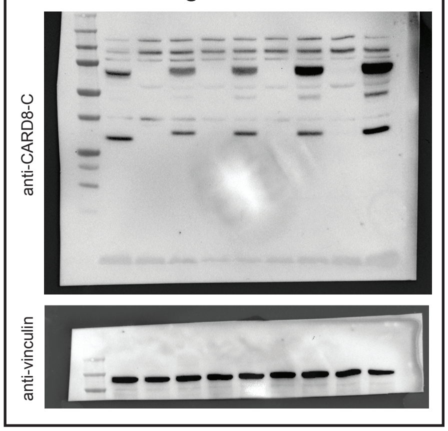

Main Figure Uncropped Western Blots: Multichannel images shown are a merge of chemiluminescent and colorimetric images. Dashed purple box indicates lanes used for main figure
