# Supplementary material for: A human-specific motif facilitates CARD8 inflammasome activation after HIV-1 infection
Source: eLife. 2023 Jul 7;12:e84108. doi: 10.7554/eLife.84108 (PMC10359095; doi:10.7554/eLife.84108)
Supplement: Source data 3. [file elife-84108-data3.pdf]

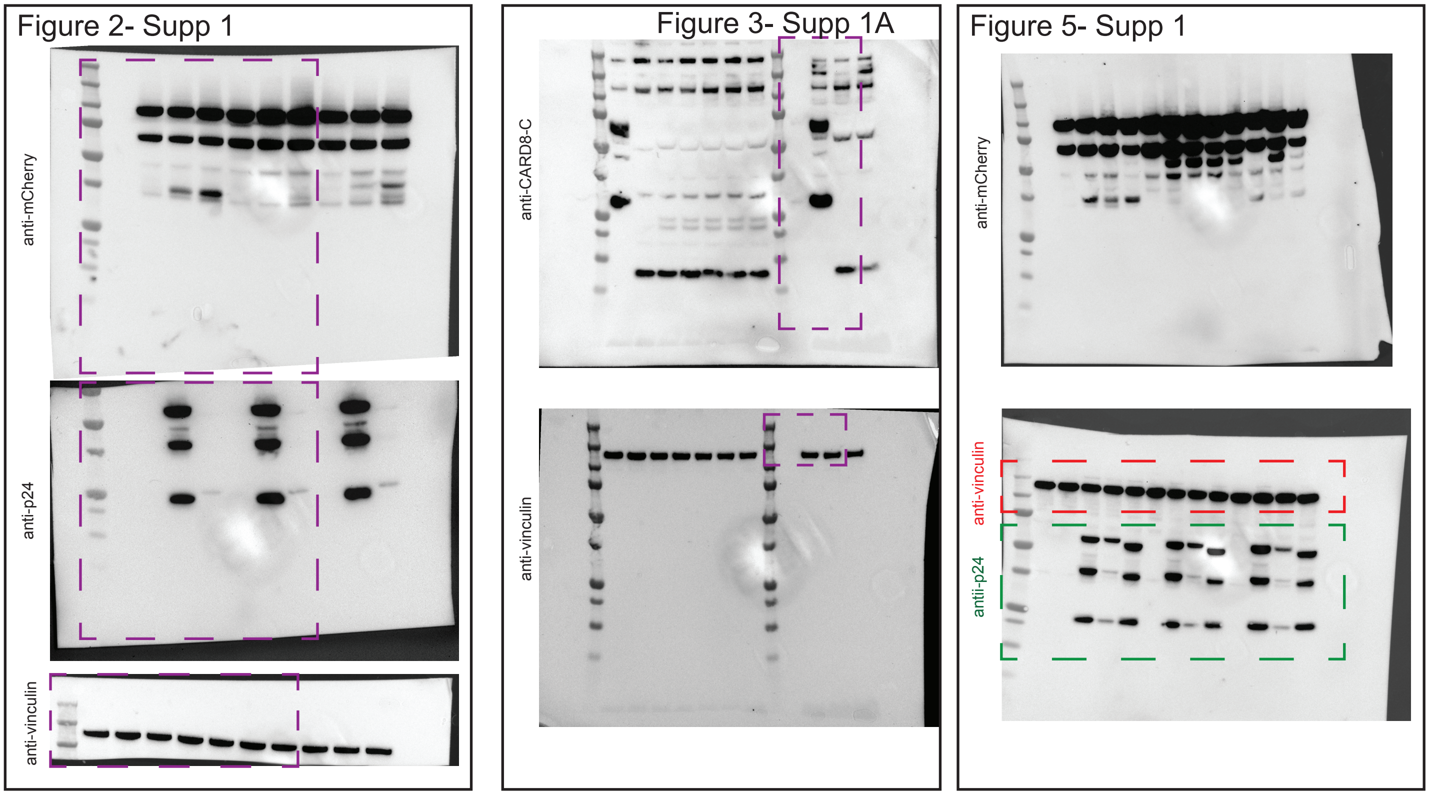

Supplemental Uncropped Western Blots:
Multichannel images shown are a merge of chemiluminescent and colorimetric images. Dashed purple box indicates lanes used for supplemental figure. Dashed red and green boxes indicate the part of the blot used for vinculin and p24 staining, respectively
